# Checklist of the fresh and brackish water snails (Mollusca, Gastropoda) of Bénin and adjacent West African ecoregions

**DOI:** 10.3897/zookeys.942.52722

**Published:** 2020-06-18

**Authors:** Zinsou Cosme Koudenoukpo, Olaniran Hamed Odountan, Bert Van Bocxlaer, Rose Sablon, Antoine Chikou, Thierry Backeljau

**Affiliations:** 1 Laboratory of Hydrobiology and Aquaculture, Faculty of Agronomic Sciences, University of Abomey-Calavi 01 BP 526 Cotonou, Bénin; 2 Biodiversité et Ressources en Eau-Benin (BioREB-ONG), 01 BP 1442, Cotonou, Bénin; 3 Laboratory of Ecology and Aquatic Ecosystem Management, Department of Zoology, Faculty of Science and Technics, University of Abomey-Calavi 01 BP 526 Cotonou, Bénin; 4 Cercle d’Action pour la Protection de l’Environnement et de la Biodiversité (CAPE BIO-ONG), Cotonou, Abomey-Calavi, Bénin; 5 Laboratory of Research on Wetlands, Department of Zoology, University of Abomey-Calavi, Abomey-Calavi, Bénin; 6 CNRS, Univ. Lille, UMR 8198 - Evo-Eco-Paleo, F-59000, Lille, France; 7 Royal Belgian Institute of Natural Sciences (RBINS), Vautierstraat 29, B-1000, Brussels, Belgium; 8 Evolutionary Ecology Group, University of Antwerp, Universiteitsplein 1, B-2610, Antwerp, Belgium

**Keywords:** biodiversity, gastropods, inland water, species inventory, West Africa

## Abstract

Currently no comprehensive checklist of fresh and brackish water gastropods from Bénin exists, and those for adjacent West African areas are outdated. Yet, such checklists provide essential biodiversity information and a consistent taxonomic and nomenclatural framework for that biodiversity. Here a first checklist of the fresh and brackish water gastropods from Bénin and adjacent West African ecoregions is presented, based on an extensive literature review and field surveys between September 2014 and June 2019 in six major fresh and brackish water ecosystems in Bénin. This inventory includes information on synonymy, species distribution in West Africa, habitats, and conservation status. The fresh and brackish water gastropod fauna includes 60 species, belonging to 28 genera and 16 families. Pachychilidae, Ampullariidae, Neritidae, and Bulinidae were the most diverse families with 9, 8, 7, and 7 species, respectively. However, literature and field data indicated that 23 species observed in West African basins that extend to Bénin do not occur in the territory of Bénin. These species were not detected in our field surveys, most likely because they are rare at collecting sites. Of the 60 species included, five are classified as “Data Deficient”, 43 as “Least Concern”, two as “Nearly Threatened”, one as “Vulnerable”, and six as “Endangered” by the IUCN, whereas the remaining three species were not evaluated. Because the taxonomy of fresh and brackish water gastropods in West Africa is still largely based on morphology, comparative molecular and taxonomic studies may result in substantial revisions of this checklist over the coming years.

## Introduction

Mollusca are the second largest animal phylum on Earth, after Arthropoda, and comprise estimated numbers of 50,000–55,000, 25,000–30,000 and 6,000–7,000 of described and valid marine, terrestrial and freshwater species, respectively (Strong et al. 2008; MolluscaBase 2019b). The largest molluscan class, Gastropoda (83% of accepted mollusc species), has repeatedly and successfully colonized continental waters on all continents, except Antarctica (Strong et al. 2008; MolluscaBase 2019b). Despite their economic interest and ecological importance in many aquatic ecosystems (Wanninger and Wollesen 2019), our understanding of their biodiversity is far from complete, especially in developing countries, where expertise, resources and facilities for biodiversity studies are limited (Odountan et al. 2019a). A poor understanding of the biodiversity that underpins ecosystems and their functioning, hampers sustainable management. Indeed, as much legislative work depends on a validated overview of taxonomic biodiversity (Araujo and Jong 2015), biodiversity inventories are essential for the development of monitoring strategies and conservation policies. Moreover, with the growing need to understand natural resources and heritage, biodiversity checklists and databases have become essential tools facilitating communication between taxonomists, naturalist data managers, ecologists, geneticists, museum curators, conservationists, etc. Beyond consolidating taxonomic knowledge, they enable study and management at organismal and ecosystem level, making them essential for national and international conservation (Lydeard et al. 2004; Régnier et al. 2009). As result, there is an increasing demand from policy makers and managers to readily have access to datasets regarding biodiversity (Gofas et al. 2017).

Malacological investigations of fresh and brackish waters are uncommon in West Africa in general and in Bénin in particular. Adanson (1757) and Dautzenberg (1912) investigated the malacological fauna of Senegal and West Africa, respectively, but their works focussed mainly on shells of marine species. The freshwater gastropods of Bénin were studied for the first time by Germain (1917), based on collections by Henry Hubert made around the 1910s. The first identification guide of West African molluscs (from Mauritania to Angola) was published in 1950 (Nicklès 1950), but focused mainly on marine taxa. Towards the end of the 20^th^ century, several malacological studies have been undertaken on freshwater and brackish taxa in West Africa and in Bénin (Sellin et al. 1980; Danish Bliharziasis Laboratory 1981; Maslin and Bouvet 1986; Zabi and Le Loeuff 1992, 1993; Brown and Kristensen 1993; Le Loeuff and Zabi 1993). These regions were also covered in the first treatise on African freshwater snails (including some considerations on brackish species) on a continental scale (Brown 1980, 1994). These taxonomic papers are now becoming outdated, and in several respects inaccurate. Indeed, since the overviews by Brown (1980, 1994) much taxonomic and faunistic progress has been made (e.g., Jørgensen et al. 2008; Hayes et al. 2015), but the results of these studies have not yet been compiled in an updated overview. Moreover, most research dealing with mollusc diversity in West Africa, including Bénin, focussed on ecology (e.g., Villanueva 2004; Gnohossou 2006; Adandedjan 2012; Odountan 2017; Zinsou 2017; Koudenoukpo 2018) or the transmission of human diseases (e.g., Ibikounlé et al. 2008, 2009, 2013, 2014a, b; Agboho 2018; Onzo-Aboki et al. 2018). As a result, the taxonomic basis of many of these studies was not up to date. This also applies to the List of non-marine molluscs of Benin in Wikipedia (https://en.wikipedia.org/wiki/List_of_non-marine_molluscs_of_Benin), which is incomplete, with outdated nomenclature, it does not include synonyms, and does not provide distributional and conservation information. As such, the Wikipedia list does not inspire much confidence (Kittur et al. 2008). Hence, a new solid and comprehensive synthesis is in order. Therefore, we here provide a comprehensive taxonomic overview of the fresh and brackish water gastropods of Bénin and adjacent West African ecoregions and compile an up to date biodiversity checklist for this fauna. This checklist was developed from literature study and verified through field surveys in six major fresh and brackish ecosystems in Bénin. It provides species synonymy, distribution and habitat data for West Africa, and conservation status. As such we hope that it will act as a reference and research tool for future taxonomic and biomonitoring studies.

## Materials and methods

### Study region: Bénin and adjacent ecoregions

**Figure 1.**
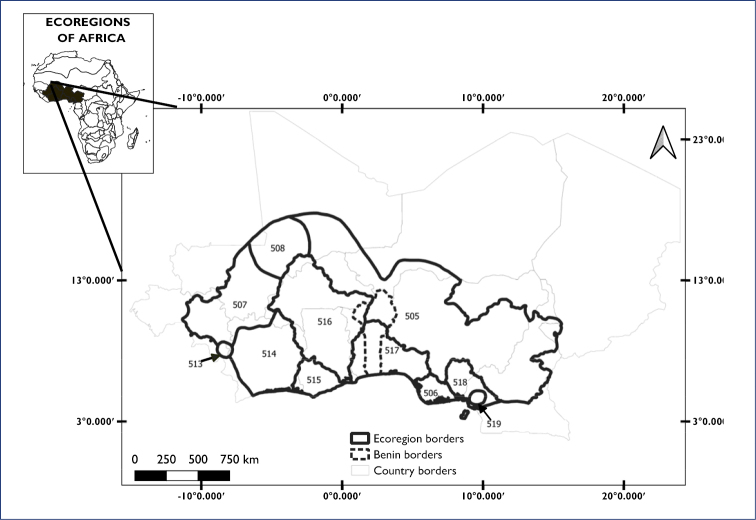
Map of Bénin and surrounding ecoregions covering the major river catchment basins. Ecoregion codes and the countries each ecoregion covers are listed in Table 1.

Bénin is located in West Africa between 6°15' and 12°25'N latitude and between 0°45' and 4°00'E longitude. Its neighbouring countries are Togo in the west, Burkina Faso in the north west, the Republic of Niger in the north (Niger River), and Nigeria in the east. In the south Bénin has a coastline of ~ 125 km along the Atlantic Ocean. Bénin extends from north to the south for ~ 700 km and its width varies between 125 km (along the coast) and 325 km (at the latitude of Tanguiéta). The country has a surface of 112.622 km² (Adam and Boko 1983) and a fairly large network of more or less permanent rivers and standing aquatic ecosystems. Generally, the rivers (e.g., Oueme River, Mono River) are modest in their flow regime and drain into the southern lentic system (e.g., Lake Nokoue, Lake Aheme). This aquatic network is subdivided into four basins, namely the Niger Basin (shared with Mauritania, Guinea, Algeria, Mali, Ivory Coast, Burkina Faso, Niger, Nigeria, Chad, and Cameroon), the Volta Basin (shared with Mali, Ivory Coast, Burkina Faso, Ghana, and Togo), the Oueme Basin (shared with Togo and Nigeria) and the Mono Basin (shared with Togo). Ecologically these watersheds also contain distinct natural communities, composed of different species with specific ecological dynamics, i.e., they represent distinct freshwater ecoregions (Abell et al. 2008; Graf and Cummings 2011). Sections of the same catchment system are sometimes subdivided into additional ecoregions, and matching freshwater ecoregions that have primarily been established for fish (Abell et al. 2008) with the Transboundary Freshwater dispute Database (https://tfddmgmt.github.io/tfdd/map.html). As such, Bénin and its immediate surroundings are covered by ecoregions 505–508 and 513–519 (Fig. 1; Table 1), which form the geographical scope of our study.

**Table 1.** Freshwater ecoregions of West Africa investigated and their attributes Ecoregions codes from Abell et al. (2008).

Ecoregions	Covered countries
505: Lower Niger–Benue	Mali, Burkina Faso, Niger, Bénin, Nigeria, Cameroon, Chad
506: Niger Delta	Nigeria,
507: Upper Niger	Guinea, Mali, Ivory Coast, Burkina Faso
508: Inner Niger Delta	Mali, Mauritania
513: Mount Nimba	Guinea, Ivory Coast
514: Eburneo	Ivory Coast, Burkina Faso
515: Ashanti	Ivory Coast, Ghana
516: Volta	Ivory Coast, Mali, Burkina Faso, Ghana, Togo, Bénin
517: Bight Drainage	Ghana, Togo, Bénin, Nigeria,
518: Northern Gulf of Guinea Drainages	Nigeria, Cameroon
519: Western Equatorial Crater Lakes	Cameroon

### Literature study

This checklist is based on a careful literature review to construct an up-to-date biodiversity inventory. These literature sources include peer-reviewed articles, books, reports, manuals, dissertations and other grey literature on the gastropods of Bénin, surrounding countries and their shared drainage basins. Indeed, the development of such a corpus of literature requires the collection of heterogeneous, sometimes contradictory, not to say conflictual, taxonomic opinions across a wide variety of publications.

### Field surveys

We supplemented the literature-based biodiversity inventory with field sampling in Bénin. Sampling was conducted in the Sô River, the Oueme River, Lake Nokoue, the Porto-Novo Lagoon, Lake Aheme and the Coastal Lagoon of Ouidah Grand-Popo. In total, 94 field excursions (24, 22, 12, 12, 12, 12, respectively), each of two days per waterbody, were organized between September 2014 and June 2019. Eight to twelve sampling sites were defined within each waterbody to cover a wide range of sub-habitats. These field surveys were performed with an Ekman grab (0.0225 m^2^) and a long-handled kick net (250 µm mesh). Specifically, we inspected the littoral area, the deeper zones, within/under aquatic macrophytes and other environments suitable for molluscs. Snails were put in formalin in prelabelled plastic containers. These containers were subsequently transported to the laboratory, where the snails were washed, and identiﬁed using appropriate identification keys (Nicklès 1950; Durand and Lévêque 1981; Brown and Kristensen 1993; Brown 1994) and compared with reference specimens from Dahomey (former name of Bénin) and Bénin (if available) deposited in the collections of the Royal Belgian Institute of Natural Sciences (RBINS) and the Royal Museum for Central Africa (RMCA).

### Data compilation

All taxa included in this study were cross-checked for their original name combination, synonymies, type locality data, habitats and dubious records against MolluscaBase (accessed at http://www.molluscabase.org during October 2019), and the Worldwide mollusc species Data Base (WMSD accessed at http://www.bagniliggia.it/ during October 2019) when MolluscaBase did not provide the required data. In addition, the conservation status of each species was determined from the IUCN red list (accessed at https://www.iucnredlist.org/ during October 2019). The discussion of the occurrence and conservation status of species whose geographical distribution extends beyond the targeted ecoregions, is mainly limited to the ecoregions covered here. We based our systematic arrangement of subclasses and orders on Bouchet et al. (2017), whereas families, genera and species are listed in alphabetical order.

## Results

Our final checklist includes 60 species belonging to 28 genera and 16 families. Information on each of these species is provided below.

Systematic Catalogue

Class GASTROPODA Cuvier, 1795

Subclass Neritimorpha Golikov & Starobogatov, 1975

Order Cycloneritida Frýda, 1998

Family NERITIDAE Rafinesque, 1815

Genus *Clypeolum* Récluz, 1842

***Clypeolum owenianum* (W. Wood, 1828)**

**Original combination.***Nerita oweniana* W. Wood, 1828.

**Synonyms.***Neritina tiassalensis* Binder, 1956.

**Type locality.** Africa (Brown 1994).

**Habitat.** Fresh and Brackish water.

**Distribution.** Ivory Coast to Cameroon, including Volta River (up to Bator) (Binder 1968; Brown and Kristensen 1993; Le Loeuff and Zabi 1993; Brown 1994; Le Loeuff 1999; Bony 2007; Kouadio et al. 2008, 2011; Edokpayi and Ikharo 2011; Diomandé et al. 2013).

**Evidence in Bénin.** Along the coast of the Atlantic Ocean (Kristensen and Stensgaard 2010e).

**IUCN status.** Least Concern.

https://www.iucnredlist.org/species/40087/10303057

**Remarks.** The last whorl encloses earlier whorls almost completely and the lip is commonly expanded in two wing-like projections which appear to be most fully developed in freshwater (Pilsbry and Bequaert 1927). The species is widely distributed and observed beyond our region of interest in countries such as Liberia, the DR Congo and Angola (Brown 1994).

Genus *Nereina* de Cristofori & Jan, 1832

***Nereina afra* (G. B. Sowerby I, 1836)**

**Original combination.***Neritina afra* G. B. Sowerby I, 1836.

**Synonyms.***Nerita africana* Récluz, 1844; *Neritina aequinoxialis* Morelet, 1848.

**Type locality.** Fernando Po (= Bioko, Equatorial Guinea).

**Habitat.** Fresh and Brackish water.

**Distribution.** From Ivory Coast to Cameroon (Brown and Kristensen 1993; Brown 1994; Bandel and Kowalke 1999; Kouadio et al. 2008, 2011).

**Evidence in Bénin.** Coastal Lagoon of Bénin (Adandedjan 2012).

**IUCN status.** Least Concern.

https://www.iucnredlist.org/species/165778/6119044

**Remarks.** Observed in our field data in ecoregion 517.

Genus *Vitta* Adams & Adams, 1854

***Vitta adansoniana* (Récluz, 1841)**

**Original combination.***Nerita adansoniana* Récluz, 1841.

**Synonyms.***Neritina adansoniana* (Récluz, 1841); *Neritina sangara* Morelet, 1848.

**Type locality.** Senegal River estuary.

**Habitat.** Fresh and Brackish water.

**Distribution.** Ivory Coast to Cameroon (Binder 1968; Brown and Kristensen 1993; Le Loeuff and Zabi 1993; Brown 1994; Guiral et al. 1999; Finlayson et al. 2000).

**Evidence in Bénin.** Presence uncertain (Kristensen and Stensgaard 2010d) and it was not found in the field surveys.

**IUCN status.** Least Concern.

https://www.iucnredlist.org/species/165788/6126163

***Vitta cristata* (Morelet, 1864)**

**Original combination.***Neritina cristata* Morelet, 1864.

**Synonyms.** -.

**Type locality.** Como River, Gabon.

**Habitat.** Fresh and Brackish water.

**Distribution.** Sierra-Leone, Ivory Coast, Cameroon and Gabon (Binder 1968; Brown and Kristensen 1993; Le Loeuff and Zabi 1993; Brown 1994; Guiral et al. 1999; Le Loeuff 1999; Kouadio et al. 2008, 2011).

**Evidence in Bénin.** Porto-Novo Lagoon, Coastal lagoon of Ouidah Grand-Popo (Adandedjan 2012; Odountan 2017).

**IUCN status.** Least Concern.

https://www.iucnredlist.org/species/14627/4450516

**Remarks.** Observed in our field data in ecoregion 517.

***Vitta glabrata* (G. B. Sowerby II, 1849)**

**Original combination.***Neritina glabrata* G. B. Sowerby II, 1849.

**Synonyms.***Clithon glabrata* (G. B. Sowerby II, 1849); *Clithon glabratum* (G. B. Sowerby II, 1849).

**Type locality.** Unknown.

**Habitat.** Fresh and Brackish water.

**Distribution.** Ivory Coast to Angola (Binder 1968; Sankaré and Etien 1991; Brown and Kristensen 1993; Le Loeuff and Zabi 1993; Guiral et al. 1999; Le Loeuff 1999; Kouadio et al. 2008, 2011; Olomukoro and Azubuike 2009).

**Evidence in Bénin.** Lake Nokoue, Porto-Novo lagoon, Coastal lagoon (Adandedjan 2012; Odountan 2017; Koudenoukpo 2018).

**IUCN status.** Least Concern.

https://www.iucnredlist.org/species/165780/6120407

**Remarks.** Observed in our field data in ecoregion 517. Very common in Porto-Novo lagoon.

***Vitta kuramoensis* (Yoloye & Adegoke, 1977)**

**Original combination.***Neritina kuramoensis* Yoloye & Adegoke, 1977.

**Synonyms.** -.

**Type locality.** Kuramo Water (a branch of Lagos Lagoon), Nigeria.

**Habitat.** Brackish and marine water.

**Distribution.** Ivory Coast, Ghana, Bénin, Nigeria and Gabon (Le Loeuff and Zabi 1993; Brown 1994; Guiral et al. 1999; Le Loeuff 1999; Kouadio et al. 2008, 2011; MolluscaBase 2019d).

**Evidence in Bénin.** Coastal lagoon (Adandedjan 2012).

**IUCN status.** Not Evaluated.

**Remarks.** Observed in our field data in ecoregion 517. Sometimes confused with *V. adansoniana* and some specimens identified as *V. adansoniana* may refer to *V. kuramoensis* (Brown 1994).

***Vitta rubricata* (Morelet, 1858)**

**Original combination.***Neritina rubricata* Morelet, 1858.

**Synonyms.** -.

**Type locality.** Senegambia (= Senegal).

**Habitat.** Fresh and Brackish water.

**Distribution.** Ivory Coast to Cameroon and Gabon (Binder 1968; Brown and Kristensen 1993; Le Loeuff and Zabi 1993; Brown 1994; Bandel and Kowalke 1999; Guiral et al. 1999; Le Loeuff 1999; Kouadio et al. 2008).

**Evidence in Bénin.** Not reported.

**IUCN status.** Least Concern.

https://www.iucnredlist.org/species/40090/10304117

**Remarks.** According to Brown (1994) syntypes from Morelet’s collection (Natural History Museum, London) are labelled from Calabar, Gabon and Congo. The confusion about the type locality and the possible syntypes is well-documented in Breure et al. (2018).

Subclass Caenogastropoda Cox, 1960

Grade Architaenioglossa Haller, 1890

Family AMPULLARIIDAE Gray, 1824

Genus *Afropomus* Pilsbry & Bequaert, 1927

***Afropomus balanoideus* (Gould, 1850)**

**Original combination.***Ampullaria balanoidea* Gould, 1850.

**Synonyms.***Afropomus balanoidea* (Gould, 1850).

**Type locality.** Grand Cape Mount, Liberia, Liberia (Cowie 2015).

**Habitat.** Freshwater.

**Distribution.** Sierra Leone, Liberia, Ivory Coast, Nigeria (Hubendick 1977; Brown and Kristensen 1993; Brown 1994; Asor et al. 2003; Daget 2003).

**Evidence in Bénin.** Not reported.

**IUCN status.** Near Threatened.

http://dx.doi.org/10.2305/IUCN.UK.2010-3.RLTS.T165386A6011118.en

**Remarks.** Present in neighbouring countries of Bénin (Binder 1963:13), it may occur in Bénin, where its preferred habitats such as ditches, creeks, and small rivers have been surveyed to limited extent only (Brown 1994). As an intermediate host of pulmonary paragonimiasis, the taxon may be of interest to human disease investigators. *Afropomus balanoides* is a misspelling.

Genus *Lanistes* Montfort, 1810

***Lanistes chaperi* (Kobelt, 1912)**

**Original combination.***Meladomus libycus chaperi* Kobelt, 1912.

**Synonyms.** -.

**Type locality.** Dahomey, Africae occidentalis (=Bénin).

**Habitat.** Freshwater.

**Distribution.** Reported from Bénin only (Cowie 2015).

**Evidence in Bénin.** Original description.

**IUCN status.** Not Evaluated.

**Remarks.** Two syntypes of this species exist in the Senckenberg Museum (Frankfurt am Main, Germany): SMF 7451 and 7452. The species has been described as a subspecies of *L. libycus*, and because of that reason it is neither specifically mentioned by Brown and Kristensen (1993), nor by Brown (1994). However, it was considered to be a valid species by Pilsbry and Bequaert (1927), which is maintained by Cowie (2015). Therefore, its ecology should be investigated further.

***Lanistes libycus* (Morelet, 1848)**

**Original combination.***Ampullaria libyca* Morelet, 1848.

**Synonyms.***Meladomus libycus* (Morelet, 1848); Meladomus (Lanistes) libycus var. albersi Kobelt, 1912; *Meladomus boettgeri* Kobelt, 1912.

**Type locality.** Gabon.

**Habitat.** Freshwater.

**Distribution.** Coastal countries of West Africa, i.e., Ivory Coast, Ghana, Togo, Bénin, Nigeria, Cameroon, Equatorial Guinea and Gabon (Brown 1994; Diomandé et al. 2009; Jørgensen et al. 2010a; Salawu and Odaibo 2014; Diakité et al. 2017; Danladi et al. 2019).

**Evidence in Bénin.**RMCA nos. 37061 and 37066 (Dahomey; ex. coll. Putzeys 1935).

**IUCN status.** Least Concern.

http://dx.doi.org/10.2305/IUCN.UK.2010-3.RLTS.T175137A7110785.en

**Remarks.** This species was not recorded during our sampling in Bénin, although it should occur in the eastern regions of Bénin, around Ketou, Pobè and Sakété. These localities are in close vicinity to Yewa North in Nigeria, where the species is abundant (Salawu and Odaibo 2014).

***Lanistes ovum* Troschel, 1845**

**Original combination.**Lanistes (Meladomus) ovum Troschel, 1845.

**Synonyms.**Lanistes (Meladomus) procerus von Martens, 1866; *Lanistes procerus* von Martens, 1866-; Lanistes ovum var. elatior von Martens, 1866; Lanistes olivaceus var. procerus von Martens, 1866; Lanistes ellipticus var. luapulensis Furtado, 1886; Lanistes affinis var. manyarana Sturany, 1894; Lanistes ovum var. plicosus von Martens, 1897; Lanistes ovum var. lacoini Germain, 1907; Lanistes ovum var.major Germain, 1907; Lanistes procerus var. minor Germain, 1907; Lanistes (Meladomus) procerus langi Pilsbry & Bequaert, 1927; Lanistes (Meladomus) connollyi Pain, 1954; Lanistes (Meladomus) ovum mweruensis Pain, 1954.

**Type locality.** Tete, Mozambique, but paralectotypes also come from Sena, Mozambique (Köhler and Glaubrecht 2006).

**Habitat.** Freshwater.

**Distribution.** Scattered localities over a large area in Africa including all West African countries (Brown 1994; Albrecht et al. 2018; Ouedraogo et al. 2018).

**Evidence in Bénin.** Alibori River (Agblonon Houelome et al. 2017).

**IUCN status.** Least Concern.

http://dx.doi.org/10.2305/IUCN.UK.2010-3.RLTS.T165799A6134027.en

**Remarks.** Specimens of *L. ovum* have been included in molecular studies (Jørgensen et al. 2008; Schultheiß et al. 2009), which suggested that multiple evolutionary lineages have been lumped into this taxon. Given that the type locality of *L. ovum* is in Mozambique, it is likely that the West African specimens resembling *L. ovum* belong to a distinct taxon. Molecular work is required to resolve the issue.

***Lanistes varicus* (O. F. Müller, 1774)**

**Original combination.***Helix varica* O. F. Müller, 1774.

**Synonyms.***Ampullaria olivacea* Lamarck, 1816; *Lanistes olivaceus* (Lamarck, 1816); *Ampullaria guinaica* Lamarck, 1822; *Meladomus adansoni* Kobelt, 1911; *Lanistes adansoni* (Kobelt, 1911); *Lanistes millestriatus* Pilsbry & Bequaert, 1927.

**Type locality.** Unknown.

**Habitat.** Freshwater (permanent and temporary).

**Distribution.** Senegal, Gambia, Mali, Ivory Coast, Ghana, Burkina Faso, Niger and Nigeria (Brown 1994).

**Evidence in Bénin.** Widespread especially at Cotonou garden ASECNA, Toho-Todougba lake, Sèhouè Hlan lake and Acron (Ibikounlé et al. 2009), Cocotomey (Agboho 2018), Oueme River (Zinsou 2017), Sô River (Koudenoukpo 2018), Alibori River (Agblonon Houelome et al. 2017), Porto Novo Lagoon and Coastal lagoon of Ouidah Grand-Popo (Adandedjan 2012).

**IUCN status.** Least Concern.

http://dx.doi.org/10.2305/IUCN.UK.2010-3.RLTS.T175132A7107425.en

**Remarks.** Observed in our field data in ecoregion 517. *Lanistes varicus* is an intermediate host for non-human schistosomes and is often investigated by parasitologists (Ibikounlé et al. 2009). It is an edible species consumed by humans in Bénin (Koudenoukpo 2018). It usually is abundant in natural permanent water bodies. *Lanistes guinaicus* mutation *depressa* Germain, 1917 (513–514) from Région des Tchis, cercle de Mono and Tchaourou (misspelled as Ichaourou)] is an unavailable name because of its infrasubspecific nature (Code, Art. 45.6, Glossary) (Cowie 2015). This taxon seems to be referable to *L. varicus*, but specimens of *L. varicus* from the localities mentioned by Germain (1917) should be further studied to elucidate the status of *L. guinaicus* mutation *depressa*. *L. varicus* as in Adandedjan (2012) is a misspelling.

Genus *Pila* Röding, 1798

***Pila africana* (von Martens, 1886)**

**Original combination.***Ampullaria africana* von Martens, 1886.

**Synonyms.** -

**Type locality.** Goldküste, Abetifi (= Ghana).

**Habitat.** Freshwater.

**Distribution.** Ivory Coast and Ghana (Brown and Kristensen 1993)

**Evidence in Bénin.** Not reported.

**IUCN status.** Least Concern.

http://dx.doi.org/10.2305/IUCN.UK.2010-3.RLTS.T165375A6007169.en

**Remarks.***Pila africana* is the most common species of *Pila* in countries to the west of Bénin. A lectotype and paralectotypes at the Natural History Museum, Berlin (ZMB) have been assigned by Köhler and Glaubrecht (2006).

***Pila ovata* (Olivier, 1804)**

**Original combination.***Ampullaria ovata* Olivier, 1804.

**Synonyms.***Lanistes ovatus* (Olivier, 1804); *Ampullaria gradata* Smith, 1881; Ampullaria erythrostoma var. stuhlmanni von Martens, 1897; Ampullaria gordoni var. bukobae von Martens, 1897; Ampullaria ovata var. conglobata von Martens, 1874; Ampullaria ovata var. deckeni von Martens, 1897; Ampullaria ovata var. emini von Martens, 1897.

**Type locality.** Lac Maréotis (Egypt).

**Habitat.** Freshwater.

**Distribution.** In West Africa only known from Nigeria and Chad. Common in East Africa from Egypt to northern Mozambique (Brown 1994).

**Evidence in Bénin.** Not reported.

**IUCN status.** Least Concern.

https://www.iucnredlist.org/species/165765/6110071

**Remarks.** The Nigerian specimens identified as *Pila ampullacea* (Linnaeus, 1758) by (Gadzama 2012) seem to belong to *Pila ovata* (Olivier, 1804). Molecular work is required to examine whether *P. ovata* indeed has a very wide geographical distribution, or whether it consists of several cryptic species that have been lumped together.

***Pila wernei* (Philippi, 1851)**

**Original combination.***Ampullaria wernei* Philippi, 1851.

**Synonyms.** -.

**Type locality.** White Nile.

**Habitat.** Freshwater.

**Distribution.** In West Africa, present in Mali, Nigeria, Cameroon, Chad and doubtfully in Guinea, Ivory Coast, Burkina Faso, Ghana, Togo, Bénin and Niger (Jørgensen et al. 2010b).

**Evidence in Bénin.** Presence uncertain (Jørgensen et al. 2010b).

**IUCN status.** Least Concern.

http://dx.doi.org/10.2305/IUCN.UK.2010-3.RLTS.T175127A7104032.en

**Remarks.** This species is common in the Niger River from Mali to Nigeria and therefore could be present in Niger tributaries in Bénin, namely the Mékrou, Alibori and Sota. It seems that reports from coastal environments in West Africa are misidentifications. Köhler and Glaubrecht (2006) designated a lectotype (Museum für Naturkunde, Berlin: ZMB 1335), a paralectotype exists apparently at the Museo Nacional de Historia Natural, Santiago, Chile (MNHNCL). This species has, like *P. ovata*, a wide geographical distribution, which, however, needs to be examined with molecular data.

Family VIVIPARIDAE Gray, 1847

Genus *Bellamya* Jousseaume, 1886

***Bellamya unicolor* (Olivier, 1804)**

**Original combination.***Cyclostoma unicolor* Olivier, 1804.

**Synonyms.***Vivipara duponti* De Rochebrune, 1881; *Bellamya bellamya* Jousseaume, 1886; *Viviparus unicolor* (Olivier, 1904).

**Type locality.** Alexandria, Egypt.

**Habitat.** Freshwater.

**Distribution.** The species is widely distributed in the northern hemisphere part of sub-Saharan Africa, and along the Nile (Brown 1994). In West Africa it occurs in Burkina Faso and Nigeria (Gadzama 2012; Gadzama et al. 2015; Ouedraogo et al. 2015, 2018).

**Evidence in Bénin.** Not reported.

**IUCN status.** Least Concern.

http://dx.doi.org/10.2305/IUCN.UK.2016-3.RLTS.T98275044A84313812.en

**Remarks.** This species has been considered to be a bucket taxon that requires taxonomic revision (Schultheiß et al. 2014). The type of the genus is *B. bellamya* Jousseaume, 1886, by original designation, which is considered a synonym of *Vivipara duponti* De Rochebrune, 1881, which represents a West African form of *Bellamya unicolor* (Olivier, 1804). The type locality of *B. bellamya* is Kora, Haut-Senegal, and of *V. duponti* the Bakoy River [= Bakoye River] at Pangalla. It is possible that one or both species would prove to be genetically distinct from *B. unicolor* upon molecular examination.

Order Littorinimorpha Golikov & Starobogatov, 1975

Family ASSIMINEIDAE H. Adams & A. Adams, 1856

Genus *Assiminea* Fleming, 1828

***Assiminea hessei* Boettger, 1887**

**Original combination.***Assiminea hessei* Boettger, 1887.

**Synonyms.** -.

**Type locality.** swamp behind the English trade house at Banana, West Zaire (= Democratic Republic of Congo).

**Habitat.** Brackish water.

**Distribution.** Nigeria, DR Congo (Brown 1994).

**Evidence in Bénin.** Not reported.

**IUCN status.** Endangered.

https://www.iucnredlist.org/species/175138/7111055

**Remarks.** This salt-tolerant species is associated with mangrove habitats, and easily overlooked because of its small size (length of ~3 mm). As such, it may potentially occur elsewhere, including in mangroves in Bénin.

Family BITHYNIIDAE Gray, 1857

Genus *Gabbiella* Mandahl-Barth, 1968

***Gabbiella africana* (Frauenfeld, 1862)**

**Original combination.***Bithynia africana* Frauenfeld, 1862.

**Synonyms.***Bithynia tournieri* Binder, 1955.

**Type locality.** West Africa (without further detail).

**Habitat.** Fresh and brackish water.

**Distribution.** Mali, Ivory Coast, Togo, and doubtfully in Ghana (Kristensen and Stensgaard 2010b; Camara et al. 2012; Bony et al. 2013).

**Evidence in Bénin.** Coastal lagoon of Ouidah Grand-Popo at many sites including Alongo, and Agonnékanmè (Adandedjan 2012).

**IUCN status.** Least Concern.

http://dx.doi.org/10.2305/IUCN.UK.2010-3.RLTS.T165403A6017400.en

**Remarks.** Observed in our field data in ecoregion 517. Previous records of this species were all in freshwater habitats, whereas the specimens reported in Bénin occurred in brackish water. Brown (1994) suggested that *Bithynia tournieri* Binder, 1955 may be conspecific, which is followed here, but in the absence of molecular studies the systematics of *Gabbiella* are very poorly known. The contrast in habitat between previously recorded specimens and those from Bénin could be indicative for specific differences, but until compelling evidence indicates otherwise, we consider the Bénin specimens conspecific.

***Gabbiella tchadiensis* Mandahl-Barth, 1968**

**Original combination.***Gabbiella tchadiensis* Mandahl-Barth, 1968.

**Synonyms.** -.

**Type locality.** South East shore at Bol in Lake Chad, Chad.

**Habitat.** Freshwater.

**Distribution.** Tchad and Nigeria (Brown 1994).

**Evidence in Bénin.** Not reported.

**IUCN status.** Endangered.

http://dx.doi.org/10.2305/IUCN.UK.2010-3.RLTS.T165387A6011471.en

**Remarks.** This species occurs in the catchment of Lake Chad, including the Komadugu Yobe River. This catchment falls beyond the ecoregions under study here, but the taxon is considered to have had a more extensive Late Pleistocene-Holocene distribution in the Chad Basin (Van Damme 1984). Additionally, recent specimens have been reported also from Lake Léré on the border between Cameroon and Chad (Brown 1994), which is part of the Niger Basin and the reason for inclusion here.

Family HYDROBIIDAE Stimpson, 1865

Genus *Hydrobia* Hartmann, 1821

***Hydrobia accrensis* Connolly, 1929**

**Original combination.***Hydrobia accrensis* Connolly, 1929.

**Synonyms.** -.

**Type locality.** Quarry near Accra, Ghana.

**Habitat.** Freshwater.

**Distribution.** Ghana and Togo (Kristensen and Stensgaard 2010c).

**Evidence in Bénin.** Not reported.

**IUCN status.** Near Threatened.

http://dx.doi.org/10.2305/IUCN.UK.2010-3.RLTS.T165400A6016291.en

**Remarks.** As for Bithyniidae, the lack of knowledge on the anatomy of hydrobiid species combined with a lack of molecular studies currently hampers confident systematic placement of African Hydrobiidae (see e.g., Seddon et al. 2011).

***Hydrobia guyenoti* Binder, 1955**

**Original combination.***Hydrobia guyenoti* Binder, 1955.

**Synonyms.** -.

**Type locality.** Toupah Bay in Lagune Ebrié, Ivory Coast.

**Habitat.** Fresh and brackish water.

**Distribution.** Ivory Coast in Lagune Ebrié (Brown 1994).

**Evidence in Bénin.** Coastal lagoon (Adandedjan 2012).

**IUCN status.** Endangered.

http://dx.doi.org/10.2305/IUCN.UK.2010-3.RLTS.T165381A6009233.en

**Remarks.** This species endemic to West Africa is not mentioned by MolluscaBase but is included in WMSDB and regional reports (Smith et al. 2009; Adandedjan 2012). It may be more widespread than previously assumed. It is one the smallest species of the genus: 2.7×1.8 mm. The whorls are strongly convex with a deep suture. The central radular tooth has a single basal denticle on each side and long lateral lobes (Brown 1994).

***Hydrobia lineata* Jekelius, 1944**

**Original combination.***Hydrobia lineata* Jekelius, 1944.

**Synonyms.** -.

**Type locality.** Bingerville Bay, in fresh water, Ivory Coast.

**Habitat.** Freshwater.

**Distribution.** Ivory Coast, Togo and Bénin (Brown 1994).

**Evidence in Bénin.** Reported in Lac Toho Todougba (Brown 1994).

**IUCN status.** Data Deficient.

http://dx.doi.org/10.2305/IUCN.UK.2010-3.RLTS.T165380A6008870.en

**Remarks.** Observed in our field data in ecoregion 517. Only one specimen was observed and that was empty shells, not a living specimen. The species is known from fossils only according to MolluscaBase (2019c), but some authors reported extant specimens (Smith et al. 2009; Badahoui et al. 2010). The species requires taxonomical study (Seddon et al. 2011).

Family LITTORINIDAE Children, 1834

Genus *Littoraria* Gray, 1833

***Littoraria angulifera* (Lamarck, 1822)**

**Original combination.***Phasianella angulifera* Lamarck, 1822.

**Synonyms.***Littorina angulifera* (Lamarck, 1822).

**Type locality.** Unknown.

**Habitat.** Brackish and marine and water.

**Distribution.** Senegal, Sierra Leone, Liberia, Ghana and Nigeria (Rosewater 1981).

**Evidence in Bénin.** Cotonou (Rosewater 1981).

**IUCN status.** Not Evaluated.

**Remarks.** Observed in our field data in ecoregion 517. Specimens from mangroves of the Coastal lagoon of Ouidah, Grand-Popo that have been assigned to *Littoraria scabra* (Linnaeus, 1758) by Adandedjan et al. (2012) seem to be referable to *Littoraria angulifera* (Lamarck, 1822). *Littoraria scabra* is very polymorphic, but endemic to the Indo-West Pacific region (Reid et al. 2010).

Subcohort Cerithiimorpha Golikov & Starobogatov, 1975

**Remarks.** A temporary order named Caenogastropoda has been established (MolluscaBase 2019a) to group a number of superfamilies that belong to the Subclass Caenogastropoda but not to the Order Littorinimorpha. This group has previously been referred to as the subcohort Cerithiimorpha (Bouchet et al. 2017), which is followed here. We do not use the temporary Order [unassigned] Caenogastropoda to avoid confusion with the Subclass Caenogastropoda.

Family PACHYCHILIDAE Fischer & Crosse, 1892

Genus *Potadoma* Swainson, 1840

***Potadoma angulata* Thiele, 1928**

**Original combination.***Potadoma angulata* Thiele, 1928.

**Synonyms.** -

**Type locality.** Samanga (known as Sanaga River), Cameroon.

**Habitat.** Freshwater.

**Distribution.** Currently, this species has only been recorded from Cameroon (Brown 1994).

**Evidence in Bénin.** Not reported.

**IUCN status.** Endangered.

https://www.iucnredlist.org/species/184556/8292306

**Remarks.** This endemic species of Cameroon is known only from the southernmost parts of ecoregion 505.

***Potadoma bicarinata* Mandahl-Barth, 1967**

**Original combination.***Potadoma bicarinata* Mandahl-Barth, 1967.

**Synonyms.** -.

**Type locality.** Volta River at Asikoko village near Frankadua, Ghana.

**Habitat.** Freshwater.

**Distribution.** Currently, this species has only been recorded from Ghana (Mandahl-Barth 1967).

**Evidence in Bénin.** Unconfirmed, may be reported by Adandedjan (2012) under *Potadoma* sp.

**IUCN status.** Least Concern.

http://dx.doi.org/10.2305/IUCN.UK.2010-3.RLTS.T165383A6009945.en

**Remarks.** The distribution of *Potadoma* is disjunct, most taxa occur in West Africa, whereas some others in Central Africa (Brown 1994). The phylogenetic affinities of taxa from both regions are currently unknown. Especially the West African taxa, several of which occur in the ecoregions under study here, display high morphological disparity. Many of the endemic *Potadoma* species from Cameroon fall just beyond the boundaries of the ecoregions considered here, such as *P. zenkeri* (von Martens, 1901). Late Cenozoic fossils suggest that the genus was more widespread before, including in the Albertine Rift (e.g., Van Damme and Pickford 2003; Salzburger et al. 2014), the Turkana Basin (Van Bocxlaer et al. 2008) and even in Botswana (Riedel et al. 2009).

***Potadoma freethi* (Gray, 1831)**

**Original combination.***Melania freethi* Gray, 1831.

**Synonyms.***Melania foenaria* Reeve, 1860; *Melania guineensis* Reeve, 1860; *Potadoma freethi dykei* Spence, 1925; *Melania nigrita* Morelet, 1851; *Melania nigritina* Morelet, 1848; *Potadoma freethii guineensis* Pilsbry & Bequaert, 1927.

**Type locality.** Fernando Po (= Bioko, Equatorial Guinea).

**Habitat.** Freshwater.

**Distribution.** From Ivory Coast to Nigeria (Brown 1994; Owojori et al. 2006; Kouadio et al. 2008).

**Evidence in Bénin.** Reported by Kristensen and Stensgaard (2010e).

**IUCN status.** Least Concern.

https://www.iucnredlist.org/species/175120/7099504

**Remarks.***P. freethi* is the type species of the genus, by original designation (Gray 1847). Observed in our field data in ecoregion 517. Several subspecies, such as *P. f. dykei* Spence, 1925 and *P. f. guineensis* Reeve, 1860 have been described and these are included here. Two other subspecies have been described from Central Africa (DR Congo), i.e., *P. f. tigrina* Connolly, 1938 and *P. f. graptoconus* Pilsbry & Bequaert, 1927, but it seems doubtful these would belong to *P. freethi* given the disjunct distribution of the genus *Potadoma*. *Melania conulus* Lea & Lea, 1851, is another species described from Fernando Po of which the original description is similar to *P. freethi*, but more research is required before we can confirm it to be a synonym. Therefore, *M. conulus* is considered to be a “taxon inquirendum” (MolluscaBase 2019a: taxon 1115355).

***Potadoma liberiensis* (Schepman, 1888)**

**Original combination.***Melania liberiensis* Schepman, 1888.

**Synonyms.***Melania sancti-pauli* Schepman, 1888; *Potadoma bequaerti* Binder, 1963.

**Type locality.** St Paul’s River near Bavia, Liberia.

**Habitat.** Freshwater.

**Distribution.** Guinea, Liberia and Ivory Coast (Mandahl-Barth 1967; Diomandé et al. 2009).

**Evidence in Bénin.** Perhaps included in *Potadoma* sp. reported by Adandedjan (2012).

**IUCN status.** Data Deficient.

http://dx.doi.org/10.2305/IUCN.UK.2010-3.RLTS.T165385A6010770.en

**Remarks.** The synonyms concern variants in which spiral ridges are developed to variable extent.

***Potadoma moerchi* (Reeve, 1859)**

**Original combination.***Melania moerchi* Reeve, 1859.

**Synonyms.** -.

**Type locality.** ‘Guinea danica’ according to Brot (1874), confirmed as Ghana by Pilsbry and Bequaert 1927.

**Habitat.** Freshwater.

**Distribution.** Ghana, Togo, Bénin and South-West Nigeria (Mandahl-Barth 1967; Salawu and Odaibo 2014).

**Evidence in Bénin.** Reported by Brown (1994).

**IUCN status.** Least concern.

http://dx.doi.org/10.2305/IUCN.UK.2010-3.RLTS.T165382A6009591.en

**Remarks.** Observed in our field data in ecoregion 517.

***Potadoma nyongensis* Spence, 1928**

**Original combination.***Potadoma nyongensis* Spence, 1928.

**Synonyms.** -.

**Type locality.** Nyong River at 3°35'N, 10°10'E, Cameroon.

**Habitat.** Freshwater.

**Distribution.** Currently, the species is only recorded from its type locality and the Man River at Sakbayeme Cameroon (Brown 1994).

**Evidence in Bénin.** Not reported.

**IUCN status.** Endangered.

https://www.iucnredlist.org/species/184695/8315726

**Remarks.***Potadoma nyongoensis*, as in MolluscaBase, is probably a misspelling. The type locality of this endemic species of Cameroon falls beyond the boundaries of the ecoregions considered here, but Man River at Sakbayeme is part of our study area.

***Potadoma togoensis* Thiele, 1928**

**Original combination.***Potadoma togoensis* Thiele, 1928.

**Synonyms.** -.

**Type locality.** White Volta River at Apaso, Ghana.

**Habitat.** Freshwater.

**Distribution.** Ghana and Togo (Brown 1994).

**Evidence in Bénin.** Perhaps included in *Potadoma* sp. reported by Adandedjan (2012).

**IUCN status.** Data Deficient.

http://dx.doi.org/10.2305/IUCN.UK.2010-3.RLTS.T165394A6014033.en

**Remarks.** Observed in our field data in ecoregion 517.

***Potadoma trochiformis* (Clench, 1929)**

**Original combination.***Goodrichia trochiformis* Clench, 1929.

**Synonyms.** -.

**Type locality.** Man River near Sakbayeme (NE of Edea), Cameroon.

**Habitat.** Freshwater.

**Distribution.** Currently, the species is reported only from its type locality (Brown 1994).

**Evidence in Bénin.** Not reported.

**IUCN status.** Endangered.

https://www.iucnredlist.org/species/184704/8318057

**Remarks.** Possibly synonymous with *P. nyongensis* (see Mandahl-Barth 1967; Brown 1994).

***Potadoma vogeli* Binder, 1955**

**Original combination.***Potadoma vogeli* Binder, 1955.

**Synonyms.** -.

**Type locality.** Agnéby (river or stream) at Abgoville, Ivory Coast.

**Habitat.** Freshwater.

**Distribution.** Ivory Coast (Brown 1994).

**Evidence in Bénin.** Not reported.

**IUCN status.** Vulnerable.

http://dx.doi.org/10.2305/IUCN.UK.2010-3.RLTS.T165393A6013707.en

Family PALUDOMIDAE Stoliczka, 1868

Genus *Cleopatra* Troschel, 1856

***Cleopatra bulimoides* (Olivier, 1804)**

**Original combination.***Cyclostoma bulimoides* Olivier, 1804.

**Synonyms.***Paludina senegalensis* Morelet, 1860; *Cleopatra pirothi* Jickeli, 1881; Cleopatra bulimoides var. richardi Germain, 1911; Cleopatra bulimoides var. welwitschi von Martens, 1897.

**Type locality.** Kalidje Canal near Alexandria, Egypt.

**Habitat.** Freshwater.

**Distribution.** In West Africa this species occurs in Senegal, Guinea, Mali, Ivory Coast, Burkina Faso, Ghana, Togo, Bénin, Niger, Nigeria, and Chad (Brown 1994; Kristensen and Stensgaard 2010a), it also occurs in Northeast Africa, including the northern part of the East African Rift.

**Evidence in Bénin.** Observed during our field data in ecoregion 517.

**IUCN status.** Least Concern.

https://www.iucnredlist.org/species/175131/7106773

**Remarks.** Observed in our field data in ecoregion 517. *Cleopatra bulimoides* is conchologically a highly polytypic species, with many nominal species in possible synonymy, such as *C. cyclostomoides* (Küster, 1852) and *C. congener* Preston, 1913. The species boundaries of *C. bulimoides* need to be explored by molecular methods.

Genus *Pseudocleopatra* Thiele, 1928

***Pseudocleopatra togoensis* Thiele, 1928**

**Original combination.***Pseudocleopatra togoensis* Thiele, 1928.

**Synonyms.** -.

**Type locality.** Volta River near Apaso, Ghana (in Togo according to Thiele, but apparently in SE Ghana near Akwamu).

**Habitat.** Freshwater.

**Distribution.** Ghana (Brown 1994).

**Evidence in Bénin.** Not reported.

**IUCN status.** Least Concern.

http://dx.doi.org/10.2305/IUCN.UK.2010-3.RLTS.T165404A6017727.en

**Remarks.***Pseudocleopatra togoensis* is the type species of the genus.

***Pseudocleopatra voltana* Mandahl-Barth, 1973**

**Original combination.***Pseudocleopatra voltana* Mandahl-Barth, 1973.

**Synonyms.** -.

**Type locality.** Volta River at Daboya, Ghana.

**Habitat.** Freshwater.

**Distribution.** Ghana (Brown 1994).

**Evidence in Bénin.** Not reported.

**IUCN status.** Data Deficient.

http://dx.doi.org/10.2305/IUCN.UK.2010-3.RLTS.T165376A6007457.en

Family POTAMIDIDAE H. Adams & A. Adams, 1854

Genus *Tympanotonos* Schumacher, 1817

***Tympanotonos fuscatus* (Linnaeus, 1758)**

**Original combination.***Murex fuscatus*Linnaeus, 1758.

**Synonyms.***Murex radula*Linnaeus, 1758; *Murex fuscatus radula*Linnaeus, 1758 ; *Nerita aculeata* O. F. Müller, 1774; *Tympanotonos radula* (Linnaeus, 1758); *Murex terebella* Gmelin, 1791; *Potamides granulatus* (Lamarck, 1816).

**Type locality.** ‘M. Mediterraneo’, an incorrect reference to the Mediterranean Sea (Brown 1994).

**Habitat.** Brackish water.

**Distribution.** Senegal to Angola (Brown and Kristensen 1993; Brown 1994)

**Evidence in Bénin.** Sô River, Coastal lagoon of Ouidah Grand-Popo, Lake Aheme, Lake Nokoue, Porto-Novo Lagoon (Gnohossou 2006; Adandedjan 2012; Odountan 2017; Koudenoukpo 2018).

**IUCN status.** Least Concern.

http://dx.doi.org/10.2305/IUCN.UK.2010-3.RLTS.T165803A6137267.en

**Remarks.** Observed in our field data in ecoregion 517. *Tympanotonus and Tympanotomus* are very common misspellings and ill-founded emendations of the genus name *Tympanotonos* (Pilsbry & Bequaert, 1927).

Family THIARIDAE Gill, 1871(1823)

Genus *Melanoides* Olivier, 1804

***Melanoides manguensis* (Thiele, 1928)**

**Original combination.***Melania manguensis* Thiele, 1928.

**Synonyms.** -.

**Type locality.** Oti River at Mangu, East Ghana (located by Thiele in Togo).

**Habitat.** Freshwater.

**Distribution.** Ghana and Ivory Coast (Brown 1994)

**Evidence in Bénin.** Not reported.

**IUCN status.** Data Deficient.

http://dx.doi.org/10.2305/IUCN.UK.2010-3.RLTS.T165379A6008515.en

**Remarks.** Presence of this species in Togo is uncertain (Brown 1994).

***Melanoides tuberculata* (O. F. Müller, 1774)**

**Original combination.***Nerita tuberculata* O. F. Müller, 1774.

**Synonyms.**Melania (Melanoides) tuberculata (O. F. Müller, 1774); *Melania tuberculata* (O. F. Müller, 1774); *Melanoides tuberculata tuberculata* (O. F. Müller, 1774); Melanoides (Melanoides) tuberculata (O. F. Müller, 1774); Melanoides (Melanoides) tuberculata tuberculata (O. F. Müller, 1774); *Melanoides tuberculatus* (O. F. Müller, 1774); *Striatella tuberculata* (O. F. Müller, 1774); *Melanoides fasciolata* Olivier, 1804; *Turritella tuberculata* Link, 1807; *Turritella turricula* Link, 1807; *Melania cancellata* Say, 1829; *Melania mauriciae* Lesson, 1831; *Melania terebra* Lesson, 1831; *Melania trunculata* Lamarck, 1822; *Melania virgulata* Quoy & Gaimard, 1834; *Melania ornata* von dem Busch, 1842; *Melania flammigera* Dunker, 1844; *Melania rivularis* Philippi, 1847; *Melania suturalis* Philippi, 1847; *Melania rustica* Mousson, 1857; *Melania commersoni* Morelet, 1860; *Melania inhambanica* von Martens, 1860; *Melania zengana* Morelet, 1860; *Melania dominula* Tapparone Canefri, 1883; *Melania flyensis* Tapparone Canefri, 1883; *Melania pellicens* Tapparone Canefri, 1883; *Melania singularis* Tapparone Canefri, 1883; *Melania baldwini* Ancey, 1899; *Thiara baldwini* (Ancey, 1899); *Melania tuberculata* var. victoriae Dautzenberg, 1908; *Melania carica* Oppenheim, 1919; *Melania dadiana* Oppenheim, 1919; Melanoides (Melanoides) carica (Oppenheim, 1919); Melanoides (Melanoides) dadiana (Oppenheim, 1919); *Melanoides tuberculata dadiana* (Oppenheim, 1919); Melanoides tuberculata var. dautzenbergi Pilsbry & Bequaert, 1927.

**Type locality.** Coromandel coast, India.

**Habitat.** Freshwater.

**Distribution.** Widespread in West Africa including Ivory Coast, Burkina Faso, Ghana, Niger, Bénin and Nigeria (Brown 1994; Gadzama 2012; Gadzama et al. 2015; Diakité et al. 2017).

**Evidence in Bénin.** Widespread in Bénin at freshwater sites including in Coastal lagoon of Ouidah Grand-Popo around Aho Channel (Adandedjan 2012), Nokoue lake and Porto-Novo Lagoon around Totchè Channel (Gnohossou 2006; Adandedjan 2012; Odountan et al. 2019b), Acron and Djidja (Agboho 2018), Pehunco town (Ibikounlé et al. 2014b), Alibori River (Agblonon Houelome et al. 2017), Oueme River (Zinsou 2017), Lake Ahémé around Tohonou (Odountan 2017).

**IUCN status.** Least Concern.

http://dx.doi.org/10.2305/IUCN.UK.2018-2.RLTS.T155675A120117210.en

**Remarks.** Observed in our field data in ecoregion 517. The taxonomic status of the parthenogenetic *M. tuberculata* is problematic, first because it contains African and Oriental strains, and the species has invaded many tropical freshwater habitats around the globe. Native and invasive strains both occur in West Africa (Van Bocxlaer et al. 2015).

***Melanoides voltae* (Thiele, 1928)**

**Original combination.***Melania voltae* Thiele, 1928.

**Synonyms.** -.

**Type locality.** Volta River at Apaso, Ghana.

**Habitat.** Freshwater.

**Distribution.** Ghana and Nigeria (Brown 1994; Mafiana and Beyioku 1998).

**Evidence in Bénin.** Not reported.

**IUCN status.** Least Concern.

http://dx.doi.org/10.2305/IUCN.UK.2010-3.RLTS.T165406A6018396.en.

Genus *Pachymelania* E. A. Smith, 1893

***Pachymelania aurita* (O. F. Müller, 1774)**

**Original combination.***Nerita aurita* O. F. Müller, 1774

**Synonyms.***Strombus tympanorum africanus* Chemnitz, 1786; *Melania zonata* Philippi, 1848; *Melania balteata* Philippi, 1851; *Melania aurita* Reeve, 1860; *Melania histrionica* Reeve, 1860; *Io rota* Reeve, 1860; *Melania subaurita* Brot, 1868; *Melania soriculata* Morelet, 1864; *Claviger auritus* Brot, 1874; *Clavigerina aurita* von Martens, 1903.

**Type locality.** Unknown.

**Habitat.** Brackish water.

**Distribution.** Senegal to Angola including Ivory Coast, Togo, and Nigeria (Binder 1968; Brown 1994; Imoobe 2008; Tampo 2015).

**Evidence in Bénin.** At coastal area of the Coastal lagoon of Ouidah Grand-Popo (Adandedjan 2012); Oueme River (Zinsou 2017); Sô River (Koudenoukpo 2018); Lake Nokoue and Lake Aheme (Odountan 2017).

**IUCN status.** Least Concern.

http://dx.doi.org/10.2305/IUCN.UK.2010-3.RLTS.T165769A6112482.en

**Remarks.** Observed in our field data in ecoregion 517. The species is morphologically variable with respect to the number of spiral cords, threads and tubercles. A molecular systematic study of *Pachymelania* is required to assess species boundaries and morphological variation.

***Pachymelania byronensis* (W. Wood, 1828)**

**Original combination.***Strombus byronensis* W. Wood, 1828.

**Synonyms.***Melania owenii* Gray, 1831; *Melania tuberculosa* Rang, 1832; *Melania rangii* Deshayes, 1838; *Pachymelania bryoni* Smith, 1893.

**Type locality.** Coast of Upper Guinea.

**Habitat.** Freshwater.

**Distribution.** Ivory Coast to Nigeria (Brown and Kristensen 1993; Brown 1994).

**Evidence in Bénin.** Coastal lagoon of Ouidah Grand-Popo (Adandedjan 2012), Oueme River (Zinsou 2017), Sô River (Koudenoukpo 2018), Lake Nokoue and Lake Aheme (Odountan 2017).

**IUCN status.** Least Concern.

http://dx.doi.org/10.2305/IUCN.UK.2010-3.RLTS.T175140A7112397.en

**Remarks.** Observed in our field data in ecoregion 517.

***Pachymelania fusca* (Gmelin, 1791)**

**Original combination.***Murex fuscus* Gmelin, 1791.

**Synonyms.***Murex fuscatus* Maton, 1804; *Pirena granulosa* Lamarck, 1822; *Melania quadriseriata* Gray, 1831; *Melania matoni* Gray 1831; *Melania mutans* Gould, 1843; *Melania tessellata* Lea, 1850; *Melania fuscaia* Hanley, 1854–1858; *Melania fusca* Reeve, 1860; *Melania loricata* Reeve, 1860; Melania matoni var. loricata Boettger, 1885; Melania quadriseriata var. carinata Brot, 1868; *Claviger matoni* Brot, 1874; *Clavigerina fusca quadriseriata* von Martens, 1903.

**Type locality.** Unknown.

**Habitat.** Fresh and brackish water.

**Distribution.** Senegal to Angola (Brown and Kristensen 1993; Brown 1994).

**Evidence in Bénin.** Mainly at sites close to the Atlantic Ocean in Lake Nokoue and Lake Aheme (Odountan 2017), Coastal lagoon of Ouidah Grand-Popo (Adandedjan 2012), Oueme River (Zinsou 2017), Sô River (Koudenoukpo 2018).

**IUCN status.** Least Concern.

http://dx.doi.org/10.2305/IUCN.UK.2010-3.RLTS.T165779A6119724.en

**Remarks.** Observed in our field data in ecoregion 517. Like *P. aurita* this species has a very variable morphology.

Subclass Heterobranchia Burmeister, 1837

Order Ellobiida Van Mol, 1867 [see Bouchet et al. (2017) for this emendation]

Family ELLOBIIDAE L. Pfeiffer, 1854(1822)

**Remarks.** The family name was first introduced in synonymy, but is now available under art. 11.6 with the authorship determined by art. 50.7 (see Bouchet et al. 2017)

Genus *Melampus* Monfort, 1810

***Melampus liberianus* H. Adams & A. Adams, 1854**

**Original combination.***Melampus liberianus* H. Adams & A. Adams, 1854.

**Synonyms.***Melampus obovatus* H. Adams & A. Adams, 1854.

**Type locality.** Liberia.

**Habitat.** Brackish (mangrove) and marine water.

**Distribution.** River estuaries from Liberia to DR Congo, including in Ghana, Cameroon and São Thomé (Pilsbry and Bequaert 1927; Brown 1994).

**Evidence in Bénin.** Not reported.

**IUCN status.** Least Concern.

https://www.iucnredlist.org/species/175139/7111601

**Remarks.***Melampus obovatus* represents a subadult stage of *M. liberianus* (Dohrn 1878).

Superorder Hygrophila Férussac, 1822

**Remarks.** Hydrophila was originally spelled as “hygrophiles” (vernacular), subsequently latinized by Herrmannsen (1847 [in 1846–1852]: 547) and established as a suborder. Later, it was treated by Thiele (1926 [in 1925–1926]: 136) as a “Sippe” [= superfamily] but it is now considered a- Superorder (see Bouchet et al. 2017)

Family BULINIDAE Fischer & Crosse, 1880

Genus *Bulinus* O. F. Müller, 1781

***Bulinus globosus* (Morelet, 1866)**

**Original combination.***Physa globosa* Morelet, 1866.

**Synonyms.**Bulinus (Physopsis) globosus (Morelet, 1866); Isidora (Physopsis) globosa (Morelet, 1866); *Physa masakaensis* Preston, 1913; *Physopsis choziensis* Preston, 1913.

**Type locality.** Dande River (Luanda Province), Angola.

**Habitat.** Freshwater.

**Distribution.** Widespread in West Africa including Mali, Ivory Coast, Burkina Faso, Ghana, Togo, Bénin, Niger, Nigeria, Cameroon, Chad, Equatorial Guinea and Gabon (Ntonifor and Ajayi 2007; Okafor and Ngang 2008; Salawu and Odaibo 2014; Diakité et al. 2017; Abe et al. 2018; Ouedraogo et al. 2018)

**Evidence in Bénin.** Widespread, especially at Djèffa and Ganhatin (Assogba and Youssao 2002); Acron, Cotonou garden ASECNA, Djidja, Nikki, Péhunco and Pèrèrè towns, Sô Ava, Pahou, Sand quarries, and Sô Tchanhoué (Ibikounlé et al. 2009, 2013, 2014a; Agboho 2018); Alibori River (Agblonon Houelome et al. 2017); Sô River (Koudenoukpo 2018).

**IUCN status.** Least Concern.

http://dx.doi.org/10.2305/IUCN.UK.2018-2.RLTS.T99504682A120114163.en

**Remarks.** Observed in our field data in ecoregion 517. *Bulinus globosus* and *Bulinus* spp. in general are important intermediate hosts for trematode parasites. Especially parasites of the genus *Schistosoma* cause debilitating tropical diseases in humans and livestock. *Bulinus globosus* is part of the *B. africanus* species complex (Jørgensen et al. 2011). Beyond the recognition of several species complexes, our general understanding of taxonomic diversity and species relationships within *Bulinus* is still limited (see Jelnes 1986), especially within the *B. truncatus/tropicus* complex where several polyploidisation events have taken place (Jørgensen et al. 2011). *Bulinus globosus* is diploid (2n = 36) (see Jelnes 1986).

***Bulinus forskalii* (Ehrenberg, 1831)**

**Original combination.***Isidora forskalii* Ehrenberg, 1831.

**Synonyms.**Bulinus (Pyrgophysa) forskalii (Ehrenberg, 1831); Bulinus (Pyrgophysa) mariei (Crosse, 1879); *Physa apiculata* Morelet, 1867; *Physa capillacea* Morelet, 1867; *Physa clavulata* Morelet, 1867; *Physa gradata* Melvill & Ponsonby, 1898; *Physa semiplicata* Morelet, 1867; *Physa turriculata* Morelet, 1867; *Physa wahlbergi* Krauss, 1848; *Pyrgophysa mariei* Crosse, 1879.

**Type locality.** Damietta, Egypt.

**Habitat.** Freshwater.

**Distribution.** Widespread in West Africa including Mali, Ivory Coast, Burkina Faso, Ghana, Togo, Bénin, Niger, Nigeria, Cameroon, Chad, Equatorial Guinea and Gabon (Ntonifor and Ajayi 2007; Okafor and Ngang 2008; Salawu and Odaibo 2014; Diakité et al. 2017; Abe et al. 2018; Ouedraogo et al. 2018).

**Evidence in Bénin.** Widespread especially at Djèffa and Ganhatin (Assogba and Youssao 2002); Cotonou garden ASECNA, Nikki, Péhunco and Pèrèrè towns, Sô Ava, Pahou’s sand quarries, Cocotomey, Djidja, and Sô Tchanhoué (Ibikounlé et al. 2009, 2013, 2014a; Agboho 2018), Alibori River (Agblonon Houelome et al. 2017), Sô River (Koudenoukpo 2018).

**IUCN status.** Least Concern.

https://www.iucnredlist.org/species/165794/6130451

**Remarks.** Observed in our field data in ecoregion 517. *Bulinus forskali* as in Agboho (2018), is a misspelling. The *B. forskalii* species complex appears to be the most deeply split *Bulinus* species complex (Jørgensen et al. 2011). Species of this complex have a much higher spire than species of other *Bulinus* complexes (Brown 1994). Further investigation did not clarify the issue: study of its shell morphology suggests *B. jousseaumei* to be distinct (Kristensen and Christensen 1991), whereas enzyme analyses support synonymization (Jelnes 1986).

***Bulinus jousseaumei* (Dautzenberg, 1890)**

**Original combination.***Isidora jousseaumei* Dautzenberg, 1890.

**Synonyms.** -.

**Type locality.** Senegal River near Medine, Mali.

**Habitat.** Freshwater.

**Distribution.** Widespread in West Africa including Mali, Burkina Faso, Togo, Niger, and Nigeria (Brown 1994, Salawu and Odaibo 2014).

**Evidence in Bénin.** Reported (Salawu and Odaibo 2014)

**IUCN status.** Least Concern.

http://dx.doi.org/10.2305/IUCN.UK.2010-3.RLTS.T165388A6011857.en

**Remarks.** Two specimens observed in our field data in ecoregion 517 seem to be referable to *B. jousseaumei* (Dautzenberg, 1890). The species is not native in Bénin (Salawu and Odaibo 2012), but seems to be introduced. *Bulinus jousseaumei* belongs to the *B. africana* species complex, and is either a distinct species (Mandahl-Barth 1965) or a form of *B. globosus* (Wright 1961).

***Bulinus senegalensis* O. F. Müller, 1781**

**Original combination.***Bulinus senegalensis* O. F. Müller, 1781.

**Synonyms.** -.

**Type locality.** Podor, Senegal.

**Habitat.** Freshwater.

**Distribution.** Mainly Sahelian, from Guinea through the middle Niger Basin to Nigeria (Brown 1994).

**Evidence in Bénin.** Not reported.

**IUCN status.** Least Concern.

http://dx.doi.org/10.2305/IUCN.UK.2010-3.RLTS.T165398A6015514.en

**Remarks.** Being first introduced as ‘*Le Bulin*’ by Adanson (1757), *Bulinus senegalensis* is the type species of the genus. The species belongs to the *B. forskalii* species complex (Brown 1994), is diploid (2n = 36) and enzyme analyses indicated that it is distinct from *B. forskalii* (Jelnes 1986). The species occurs mainly in seasonal rain pools and aestivates during drought (Brown 1994). It was not observed in our survey of perennial waterbodies in Bénin. The species is an important host of *Schistosoma haematobium* in various regions of West Africa.

***Bulinus truncatus* (Audouin, 1827)**

**Original combination.***Physa truncata* Audouin, 1827.

**Synonyms.**Bulinus (Bulinus) truncatus (Audouin, 1827); Bulinus (Bulinus) truncatus truncatus (Audouin, 1827); Bulinus (Isidora) truncatus (Audouin, 1827); Bulinus (Isidora) truncatus truncatus (Audouin, 1827); *Physa rohlfsi* Clessin, 1886; Bulinus (Bulinus) truncatus rohlfsi (Clessin, 1886); *Bulinus rohlfsi* (Clessin, 1886); *Physa mutandaensis* Preston, 1913.

**Type locality.** Egypt.

**Habitat.** Freshwater.

**Distribution.** Widespread in West Africa including Mali, Ivory Coast, Burkina Faso, Ghana, Togo, Bénin, Niger, Nigeria, Cameroon, Chad, Equatorial Guinea, and Gabon (Ntonifor and Ajayi 2007; Okafor and Ngang 2008; Salawu and Odaibo 2014; Diakité et al. 2017; Abe et al. 2018; Ouedraogo et al. 2018).

**Evidence in Bénin.** Widespread especially at Djèffa and Ganhatin (Assogba and Youssao 2002), Accron, Cotonou garden ASECNA, Djidja, Nikki, Péhunco and Pèrèrè towns, Sô Ava, Pahou, Sand quarries, and Sô Tchanhoué (Ibikounlé et al. 2009, 2013, 2014a; Agboho 2018), Alibori River (Agblonon Houelome et al. 2017), Sô River (Koudenoukpo 2018).

**IUCN status.** Least Concern.

http://dx.doi.org/10.2305/IUCN.UK.2018-2.RLTS.T99507883A120114540.en

**Remarks.** Observed in our field data in ecoregion 517. *Bulinus truncatus* is tetraploid (2n = 72) (Jelnes 1986) and morphologically very variable (Brown 1994). Detailed studies with high-throughput sequencing are required to address many of the outstanding questions related to the biology of this species, for example, on heterozygosity and potential interspecies molecular variation across the wide geographic range of *B. truncatus*. Species such as *B. guernei* (Dautzenberg, 1890), *B. contortus* Michaud, 1829, *B. coulboisi* (Bourguignat, 1888), *B. mutandaensis* (Preston, 1913), and *B. sericinus* (Jickeli, 1874) are regularly considered to be synonyms of *B. truncatus* (Brown, 1994).

***Bulinus umbilicatus* Mandahl-Barth, 1973**

**Original combination.***Bulinus umbilicatus* Mandahl-Barth, 1973.

**Synonyms.** -.

**Type locality.** Zalingei in Darfur Province, West Sudan.

**Habitat.** Freshwater.

**Distribution.** Widespread in West Africa mainly in Mali, Niger, Nigeria, and Chad (Brown 1994).

**Evidence in Bénin.** Not reported.

**IUCN status.** Least Concern.

https://www.iucnredlist.org/species/175134/7109049

**Remarks.***Bulinus umbilicatus* is diploid (2n = 36), belongs to the *B. africanus* species complex (Jelnes 1986; Brown 1994), and displays intergradation with *B. globosus* both at the level of allozymes and shell morphology (Jelnes 1986; Kristensen and Christensen 1991). Like *B. senegalensis*, the taxon frequently occurs in seasonal aquatic habitats and aestivates during dry periods (Brown 1994).

Genus *Indoplanorbis* Annandale & Prashad, 1921

***Indoplanorbis exustus* (Deshayes, 1833)**

**Original combination.***Planorbis exustus* Deshayes, 1833.

**Synonyms.***Planorbis indicus* Benson, 1836; *Planorbis coromandelicus* Dunker, 1856; *Planorbis zebrinus* Dunker, 1856; *Planorbis hindu* Clessin, 1886; Planorbis indicus var. zonatus Clessin, 1886.

**Type locality.** marshes on the coast of Malabar, South West India.

**Habitat.** Freshwater.

**Distribution.** Ivory Coast and Nigeria (Brown 1994; Koné et al. 2013).

**Evidence in Bénin.** Freshwater habitats of Parakou city, Pahou, Sand quarry, Acron, Djassin, Djeffa, Tchivié, Cotonou, ASECNA garden, and Sô Ava (Ibikounlé et al. 2009, 2013; Agboho 2018).

**IUCN status.** Least Concern.

https://www.iucnredlist.org/species/165594/17211568

**Remarks.** Observed in our field data in ecoregion 517. Some authors have erroneously used “1834” as the year of publication. The species is native to Asia, and has been introduced into West Africa by man (Kristensen and Ogunnowof 1987). Originally, these introductions were to Ivory Coast and Nigeria, but our data suggest that the taxon is spreading in West Africa. Although *Indoplanorbis* hosts *Schistosoma* species that parasitise domestic livestock in Asia, no evidence exists to our knowledge that it transmits schistosomes in Africa.

Family LYMNAEIDAE Rafinesque, 1815

Genus *Radix* Montfort, 1810

***Radix natalensis* (Krauss, 1848)**

**Original combination.***Linnaeus natalensis* Krauss, 1848.

**Synonyms.**Lymnaea (Radix) natalensis Krauss, 1848; *Lymnaea natalensis* Krauss, 1848; Radix (Exsertiana) natalensis (Krauss, 1848); *Radix hovarum* (Tristram, 1863); *Limnaea hovarum* Tristram, 1863; Limnaeus natalensis var. exsertus von Martens, 1866; *Limnaea orophila* Morelet, 1867; *Limnaea electa* Smith, 1882; *Limnaea caillaudi* Bourguignat, 1883; *Limnaea acroxa* Bourguignat, 1883; *Lymnaea caillaudi* (Bourguignat, 1883); *Limnaea gravieri* Bourguignat, 1885; *Limnaea nyansae* von Martens, 1892; *Limnaea arabica* Smith, 1894; *Limnaea arabica* Smith, 1894; *Lymnaea arabica* Smith, 1894; *Limnaea elmeteitensis*. Smith, 1894; *Limnaea humerosa* von Martens, 1897; *Limnaea undussumae* von Martens, 1897; *Limnaeus dakaensis* Sturany, 1898.

**Type locality.** Natal, South Africa.

**Habitat.** Freshwater.

**Distribution.** Widespread in West Africa including Senegal, Burkina Faso, Ivory Coast, Nigeria (Gadzama 2012; Koné et al. 2013; Salawu and Odaibo 2014; Diakité et al. 2017).

**Evidence in Bénin.** Djèffa and Ganhatin (Assogba and Youssao 2002), Acron, Baaka, Cotonou ASECNA garden, Cotonou beach temporary ponds, Lake Nokoue, Lake Toho-Todougba, and Sèhouè bridge (Ibikounlé et al. 2009; Agboho 2018), Nikki, Pehunco and Pèrèrè towns (Ibikounlé et al. 2014a, b)

**IUCN status.** Least Concern.

http://dx.doi.org/10.2305/IUCN.UK.2018-2.RLTS.T165761A120112796.en.

**Remarks.** Observed in our field data in ecoregion 517. Some authors have erroneously used “1948” (Agboho 2018) as year of the publication of the name. The species occurs throughout most of Africa, including Madagascar, several islands in the Indian Ocean and Arabia. It usually lives in permanent waters and it is rare in seasonal habitats, unless they are directly connected to permanent waters. Molecular work is required to examine whether *R. natalensis* indeed has a very wide geographical distribution or whether it consists of several cryptic species that have been lumped together.

Family PLANORBIDAE Rafinesque, 1815

Genus *Biomphalaria* Preston, 1910

***Biomphalaria pfeifferi* (Krauss, 1848)**

**Original combination.***Planorbis pfeifferi* Krauss, 1848.

**Synonyms.**Planorbis (Coretus) pfeifferi Krauss, 1848; Planorbis (Planorbula) pfeifferi Krauss, 1848; *Biomphalaria madagascariensis* (Smith, 1882); *Planorbis hildebrandti* von Martens, 1882; *Planorbis madagascariensis* Smith, 1882; *Planorbis bowkeri* Melvill & Ponsonby, 1893; *Planorbis nairobiensis* Dautzenberg, 1908; *Planorbis hermanni* Boettger, 1910.

**Type locality.** Natal in Umgeni Valley, South Africa.

**Habitat.** Freshwater.

**Distribution.** Widespread especially in Ivory Coast, Burkina Faso Niger, Nigeria (Okafor and Ngang 2008; Salawu and Odaibo 2014; Diakité et al. 2017; Abe et al. 2018; Ouedraogo et al. 2018).

**Evidence in Bénin.** Widespread especially at Djèffa and Ganhatin (Assogba and Youssao 2002), Toho Todougba Lake, Kpinnou Lake, Sonon, Nikki, Péhunco and Pèrèrè towns, Sô Ava (Ibikounlé et al. 2009, 2013, 2014a; Agboho 2018).

**IUCN status.** Least Concern.

http://dx.doi.org/10.2305/IUCN.UK.2015.RLTS.T165782A85689765.en

**Remarks.** Observed in our field data in ecoregion 517. Enzyme studies on populations of *B. pfeifferi* from Cameroon and Senegal have found consistent biological differences (Mimpfoundi and Greer 1990), suggesting that multiple West African species of *Biomphalaria* may have been lumped in *B. pfeifferi*.

***Biomphalaria camerunensis* (Boettger, 1941)**

**Original combination.***Australorbis camerunensis* Boettger, 1941.

**Synonyms.***Biomphalaria alexandrina wansoni* Mandahl-Barth, 1957.

**Type locality.** Mongongo, NW of Mount Cameroon, Cameroon.

**Habitat.** Freshwater.

**Distribution.** From Ghana eastwards to Central African Republic (Brown 1994).

**Evidence in Bénin.** Not reported.

**IUCN status.** Least Concern.

https://www.iucnredlist.org/species/175130/7105918

**Remarks.** In Cameroon *B. camerunensis* is confined to the southern equatorial climatic zone (Greer et al. 1990), and it was never found in the same site as B. *pfeifferi* (Brown, 1994).

Genus *Gyraulus* Charpentier, 1837

***Gyraulus costulatus* (Krauss, 1848)**

**Original combination.***Planorbis costulatus* Krauss, 1848.

**Synonyms.**Planorbis (Gyraulus) costulatus Krauss, 1848; *Caillaudia angulata* Bourguignat, 1883.

**Type locality.** Natal in Umgeni Valley, South Africa.

**Habitat.** Freshwater.

**Distribution.** From Senegal to Angola including Ivory Coast, Bénin and Nigeria (Brown 1994; Salawu and Odaibo 2014).

**Evidence in Bénin.** Alibori River (Agblon Houelome et al. 2017).

**IUCN status.** Least Concern.

https://www.iucnredlist.org/species/165767/6111409

**Remarks.** Observed in our field data in ecoregion 517. The taxonomy of African *Gyraulus* is poorly known, but Meier-Brook (1983) found the African species *G. costulatus* and *G. connollyi* to have distinct anatomical characteristics that warranted him to place them in the subgenus Caillaudia Bourguignat, 1883. So far, this alternate representation has not been formally accepted.

Genus *Hovorbis* Brown & Mandahl-Barth, 1973

The genus was formerly known as *Afrogyrus* Brown and Mandahl-Barth, 1973, which however is an invalid junior homonym of the coleopteran genus *Afrogyrus* Brinck, 1955. Özdikmen and Darilmaz (2007) altered the name to *Africanogyrus*, however, the available name *Hovorbis* Brown and Mandahl-Barth, 1973 has priority.

***Hovorbis coretus* (de Blainville, 1826)**

**Original combination.***Planorbis coretus* de Blainville, 1826.

**Synonyms.***Planorbis coretus* de Blainville, 1826; *Africanogyrus coretus* (de Blainville, 1826); *Afrogyrus coretus* (de Blainville, 1826); *Planorbis misellus* Morelet, 1867; Planorbis (Spiralina) anderssoni Ancey, 1890; *Planorbis anderssoni* Ancey, 1890.

**Type locality.** Podor, Senegal.

**Habitat.** Freshwater.

**Distribution.** Bénin, Burkina Faso, Cameroon, Chad, Ivory Coast, Equatorial Guinea, Ghana, Guinea, Guinea-Bissau, Niger, Nigeria, Sierra Leone, Togo. (see www.iucnredlist.org/species/165775/120113348).

**Evidence in Bénin.** Not reported.

**IUCN status.** Least Concern.

http://dx.doi.org/10.2305/IUCN.UK.2018-2.RLTS.T165775A120113348.en

**Remarks.** Two specimens observed in our field data in ecoregion 517 seem to be referable to *H. coretus*. This species was first introduced as ‘*Le Coret*’ by Adanson (1757). Several potential synonyms are mentioned in Brown (1994), but more study of these taxa is required to verify their status.

Genus *Segmentorbis* Mandahl-Barth, 1954

***Segmentorbis angustus* (Jickeli, 1874)**

**Original combination.***Segmentina angusta* Jickeli, 1874.

**Synonyms.**Planorbis (Segmentina) emicans Melvill & Ponsonby, 1892; Segmentina (Hippeutis) emicans (Melvill & Ponsonby, 1892); *Segmentina kempi* Preston, 1912.

**Type locality.** Toquor River at Mekerka (west of Asmara) in Hamasen Province, Ethiopia.

**Habitat.** Freshwater.

**Distribution.** Ivory Coast (Diakité et al. 2017); Nigeria and Cameroon (Kristensen and Stensgaard 2010; Salawu and Odaibo 2012; Salawu and Odaibo 2014).

**Evidence in Bénin.** Not reported.

**IUCN status.** Least Concern.

https://www.iucnredlist.org/species/165771/6114438

**Remarks.***Segmentorbis angustus* is the type species of the genus. The small body size of *Segmentorbis* species (<6 mm) implies that it may be sometimes be overlooked in freshwater snail surveys. *Segmentorbis angustus* occurs in permanent waterbodies, often within the vegetation.

***Segmentorbis kanisaensis* (Preston, 1914)**

**Original combination.***Segmentina kanisaensis* Preston, 1914.

**Synonyms.***Segmentorbis formosa* Connolly, 1928.

**Type locality.** Nile at Kanisa, South Sudan.

**Habitat.** Freshwater.

**Distribution.** Widely distributed in West Africa from Gambia to Chad (Albrecht et al. 2008)

**Evidence in Bénin.** Not Reported.

**IUCN status.** Least Concern.

http://dx.doi.org/10.2305/IUCN.UK.2010-3.RLTS.T165763A6107847.en

**Remarks.** This species can be readily distinguished from *S. angustus* by its depressed shell with strongly carinated periphery. It is sometimes found together with *S. angustus*, but also occurs in temporary waters (Brown 1994).

Family PHYSIDAE Fitzinger, 1833

Genus *Afrophysa* Starobogatov, 1967

***Afrophysa brasiliensis* (Küster, 1844)**

**Original combination.***Physa brasiliensis* Küster, 1844.

**Synonyms.***Physa mosambiquensis* Clessin, 1886; Physa (Aplecta) waterloti Germain, 1911; *Aplexa waterloti* Brown, 1994.

**Type locality.** “Brasil” but Taylor (2003) restricted it to Porto Alegre, Rio Grande do Sul.

**Habitat.** Freshwater.

**Distribution.** Ghana, Togo and Nigeria (MolluscaBase 2018).

**Evidence in Bénin.** Porto-Novo (Germain 1911), Sô River (Odountan 2017; Koudenoukpo 2018).

**IUCN status.** Least Concern (evaluated under *Aplexa waterloti*).

https://www.iucnredlist.org/species/165396/6014756

**Remarks.** Observed in our field data in ecoregion 517. Physa (Aplecta) waterloti Germain, 1911 was established by Taylor (2003) as junior synonym of *Afrophysa brasiliensis* based on type specimens (from Bénin) which were morphologically degraded and very bad. Molecular work on specimens from the type locality in Brazil and West Africa is required to resolve relationships within *Afrophysa*.

Genus *Physella* Haldeman, 1842

***Physella acuta* (Draparnaud, 1805)**

**Original combination.***Physa acuta* Draparnaud, 1805.

**Synonyms.***Haitia acuta* (Draparnaud, 1805); *Lymnaea heterostropha* Say, 1817; *Physa fontana* Haldeman, 1841; *Physa inflata* Lea, 1841; *Physa charpentieri* Küster, 1850; Physa heterostropha nigricans var. callosa Rigacci, 1866; Physa heterostropha var. gibbosa Rigacci, 1866; Physa heterostropha var. minor Rigacci, 1866; *Physa lata Tryon*, 1865; *Physa plicata* De Kay, 1843; *Physa philippii* Küster, 1844; *Physa primeana* Tryon, 1865; *Physa say de* Blainville, 1826; *Physa striata* Menke, 1828; *Physa tenuissima* Lea, 1864.

**Type locality.** River Garonne, France.

**Habitat.** Freshwater.

**Distribution.** Widely distributed in West Africa from Senegal to Angola (Van Damme et al. 2017)

**Evidence in Bénin.** Acron, Cocotomey, Djeffa, and Djidja (Agboho 2018).

**IUCN status.** Least Concern.

http://dx.doi.org/10.2305/IUCN.UK.2017-3.RLTS.T155538A91354457.en

**Remarks.** Observed in our field data in ecoregion 517. The taxon is native to America and has been introduced in many other regions around the world including Europe, Asia and Africa. The African *Physella* fauna likely consists of a composite from multiple introductions. Nominal species such as *Physa borbonica* Férussac, 1827, *P. cubensis* Pfeiffer, 1839, *P. canariensis* Bourguignat, 1856, *P. tenerifae* Mousson, 1872, *P. mamoi* Benoit, 1875, and *Aplecta orbignyi* Mazé, 1883, considered as synonyms of *P. acuta* (e.g Brown 1994), are not mentioned in MolluscaBase.

Genus *Stenophysa* von Martens, 1898

***Stenophysa marmorata* (Guilding, 1828)**

**Original combination.***Physa marmorata* Guilding, 1828.

**Synonyms.**Limnea (Physa) rivalis Sowerby, 1822; *Aplexa marmorata* (Guilding, 1828); *Physa acuminata* Villa & Villa, 1841; *Aplecta sowerbyana* d’Orbigny, 1841.

**Type locality.** St. Vincent, Lesser Antilles.

**Habitat.** Freshwater.

**Distribution.** Ivory Coast (Bony et al. 2008), Nigeria (Oloyede et al. 2017).

**Evidence in Bénin.** Djèffa and Ganhatin (Assogba and Youssao 2002), Sô Ava (Ibikounlé et al. 2013).

**IUCN status.** Least Concern.

http://dx.doi.org/10.2305/IUCN.UK.2011-2.RLTS.T189786A8768994.en

**Remarks.** Observed in our field data in ecoregion 517.

## Discussion

This study provides the first checklist of fresh and brackish water gastropods in Bénin and adjacent ecoregions, i.e., ecoregions 505–508 and 513–519 of Abell et al. (2008). It comprises a total of 60 species, classified in 28 genera. More specifically, Architaenioglossa, Cerithiimorpha, Cycloneritida, Ellobiida, Hygrophila, and Littorinimorpha comprise 9, 19, 7, 1, 17, and 7 species, respectively. From the 16 families listed, Pachychilidae, Ampullariidae, Neritidae, Bulinidae, and Thiaridae were the most diverse with 9, 8, 7, 7, and 6 species, respectively. Of the 60 species listed, 37 are recorded (sometimes uncertain) in Bénin (~ 62 %), indicating a considerable species richness. The high richness in Pachychilidae relates to the diversity within the genus *Potadoma*, whereas the high richness in Ampullariidae relates to the diversity within the genera *Pila* and *Lanistes* throughout (sub-)tropical Africa (Cowie 2015). However, almost half of the Pachychilidae and one third of the ampullariids that are recorded in this study have not been recorded directly from Bénin. The fact that only a small part of Bénin’s aquatic environments, especially around the Niger River, the largest river in West Africa, have been sampled might explain why some species that are broadly distributed in West Africa such as *Pila wernei* (endemic to the Niger River basin) and *Bulinus senegalensis* O. F. Müller, 1781 have not been detected in our sampling.

Our findings with literature-based data also provoked some taxonomic concerns, because several papers on the fresh and brackish water malacofauna of Bénin or West-Africa, contained several (nomenclatural) errors. A case in point is the erroneous listing of *Codakia orbicularis* (Linnaeus, 1758), *Cardita calyculata* (Linnaeus, 1758), *Thais coronata califera* (Lamarck, 1822), *Thais nodosa* (Linnaeus, 1758), *Turritella* Lamarck, 1799, *Polinices* Montfort, 1810, *Patella*Linnaeus, 1758 as non-marine gastropod species in Bénin (e.g., Adandedjan 2012). These taxa were excluded in this study. In addition, we were unable to find information on *Melanoides anomala* (Smith, 1877) reported from Bénin (Adandedjan et al. 2012), and this taxon was consequently omitted. This identification seems to refer to *Melanoides anomala* (Dautzenberg & Germain, 1914), which has its type locality in the DR Congo and is endemic to the Congo Basin (Brown 1994). The difference in authorship and the report of the species outside its known region is suspect and calls for verification. Similarly, records that cannot be checked, e.g., *Lanistes ovum* (Agblonon Houelome et al. 2017) because specimens have not been illustrated and nor deposited in publicly accessible institutions, should be treated with caution. Hence, until compelling evidence indicates otherwise, we regard such doubtful species records in the literature as misidentifications.

Although only four species are threatened (Endangered/Vulnerable), a significant number of species has been assessed as Data Deficient, Not assessed or Not applicable. One of the main reasons for Data Deficiency in molluscs is taxonomic uncertainty and poor geographic knowledge (Seddon et al. 2011). Moreover, in West Africa, there are only few, reliable, recent survey data available, so that more species were marked as Data Deficient (Seddon et al. 2011). Therefore, a large field inventory is required that should focus on diverse habitats of fresh and brackish water from North to South with the possibility of molecular analyses. Moreover, species such as *Afrophysa brasiliensis* (Küster, 1844), *Lanistes guinaicus* mutation *depressa* Germain, 1917, *Lanistes chaperi* (Kobelt, 1912), *Lanistes ovum* Troschel, 1845, Physa (Aplecta) waterloti Germain, 1911, *Pila ovata* (Olivier, 1804), and *Radix natalensis* (Krauss, 1848) need further taxonomic study.

Bénin and its transboundary basins present a diversified fresh and brackish water gastropod fauna. The current checklist contains information on 60 species. However, many of these species require more detailed taxonomic and phylogenetic scrutiny, our current knowledge remains in its infancy. This checklist is hence an updated baseline for further taxonomic and ecological studies of the fresh and brackish water gastropods of Bénin and adjacent West African ecoregions.

## References

[B1] Abe EM Guan W Guo YH Kassegne K Qin ZQ Xu J Chen JH Ekpo UF Li SZ Zhou XN (2018) Differentiating snail intermediate hosts of *Schistosoma* spp. using molecular approaches: fundamental to successful integrated control mechanism in Africa. Infectious Diseases of Poverty 7: 1–29. 10.1186/s40249-018-0401-z 29615124 PMC5883423

[B2] Abell R Thieme ML Revenga C Bryer M Kottelat M Bogutskaya N Coad B Mandrak N Contreras Balderas S Bussing W Stiassny MLJ Skelton P Allen GR Unmack P Naseka A Ng R Sindorf N Robertson J Armijo E Higgins J V. Heibel TJ Wikramanayake E Olson D López HL Reis RE Lundberg JG Sabaj Pérez MH Petry P (2008) Freshwater Ecoregions of the World: A new map of biogeographic units for freshwater biodiversity conservation. BioScience 58: 403–414. 10.1641/B580507

[B3] Adam KS Boko M (1983) Le Bénin. SODIMAS/EDICEF, Cotonou, Benin, 95 pp.

[B4] Adandedjan D (2012) Diversité et déterminisme des peuplements de macroinvertébrés benthiques de deux lagunes du sud-Bénin: la lagune de Porto-Novo et la lagune côtière. PhD Thesis, Université d’Abomey-Calavi, Bénin.

[B5] Adandedjan D Laleye P Gourene G (2012) Macroinvertebrates communities of a Coastal Lagoon in southern Benin, West Africa. International Journal of Biological and Chemical Sciences 6: 1233–1252. 10.4314/ijbcs.v6i3.27

[B6] Adanson M (1757) Histoire Naturelle du Sénégal: Coquillages, Avec la Relation Abrégée d’un Voyage Fait en ce Pays Pendant les Années 1749, 50, 51, 52 & 53. Paris (France), 275 pp. 10.5962/bhl.title.11585

[B7] Agblonon Houelome TM Adandedjan D Chikou A Imorou Toko I Koudenoukpo C Bonou C Youssao AKI Laleye P (2017) Inventory and faunistic characteristics of the macroinvertebrates of the Alibori River in the cotton basin of Benin. International Journal of Innovation and Applied Studies 21: 433–448.

[B8] Agboho P (2018) Taxinomie, bioécologie et caractérisation moléculaire des Sciomyzidae, prédateurs de mollusques hôtes intermédiaires de bilharziose au Bénin, Afrique de l’Ouest. PhD Thesis, Université d’Abomey-Calavi, Bénin.

[B9] Albrecht C Clewing C Jørgensen A Kristensen TK Lange C Stensgaard A-S (2018) *Lanistes ovum* The IUCN Red List of Threatened Species 2018: e.T165799A120111809. 10.2305/IUCN.UK.2018-2.RLTS.T165799A120111809.en

[B10] Araujo R Jong Y De (2015) Fauna Europaea: Mollusca – Bivalvia Biodiversity Data Journal 3: e5211. 10.3897/BDJ.3.e5211 PMC4549642 26311403

[B11] Asor JE Ibanga ES Arene FOI (2003) The epidemiology of pulmonary Paragonimiasis in Cross River Basin in Nigeria: update on infection prevalence and distribution of the snail and crab intermediate hosts. Mary Slessor Journal of Medicine 3: 5–12. 10.4314/msjm.v3i1.10988

[B12] Assogba MN Youssao AKI (2002) Étude parasitologique de *Radix natalensis* (Krauss, 1848) (Gastropoda, Lymnaeidae), hôte intermédiaire de *Fasciola gigantica* (Cobbold, 1885), au Bénin. Revue de Médecine Vétérinaire 153: 407–410.

[B13] Badahoui A Fiogbe ED Boko M (2010) Les causes de la dégradation du lac Ahémé et ses chenaux. International Journal of Biological and Chemical Sciences 4: 882–897. 10.4314/ijbcs.v4i4.62971

[B14] Bandel K Kowalke T (1999) Gastropod fauna of the Cameroonian coasts. Helgoland Marine Research 53: 129–140. 10.1007/s101520050016

[B15] Binder E (1963) La reserve naturelle intégrale du Mont Nimba. I. Mollusques. Mémoires de l’Institut français d’Afrique Noire 66: 13–31.

[B16] Binder E (1968) Répartition des mollusques dans la lagune Ebrié (Côte d’Ivoire). Cahiers ORSTOM Série Hydrobiologie 2: 3–34.

[B17] Bony KY (2007) Biodiversité et écologie des mollusques gastéropodes d’eau douce en milieu continental ivoirien (bassins de la Mé, de l’Agnéby et du Banco). Traits d’histoire de vie d’une espèce invasive *Indoplanorbis exustus* (Deshayes, 1834). PhD Thesis, Ecole Pratique des Hautes Etudes, Perpignan.

[B18] Bony KY Edia EO Konan KF Kouassi n’gouan C Diomandé D Ouattara A (2013) Spatial Distribution pattern of Freshwater Mollusks in Mé, Agnéby and Banco basins (Ivory Coast; West Africa). Bulletin of Environment, Pharmacology and Life Sciences 2: 146–151.

[B19] Bony YK Kouassi NC Diomandé D Gourene G Verdoit-Jarraya M Pointier JP (2008) Ecological conditions for spread of the invasive snail *Physa marmorata* (Pulmonata: Physidae) in the Ivory Coast. African Zoology 43: 53–60. 10.3377/1562-7020(2008)43[53:ECFSOT]2.0.CO;2

[B20] Bouchet P Rocroi J-P Hausdorf B Kaim A Kano Y Nützel A Parkhaev P Schrödl M Strong EE (2017) Revised classification, nomenclator and typification of Gastropod and Monoplacophoran families. Malacologia 61: 1–526. 10.4002/040.061.0201

[B21] Breure ASH Audibert C Ablett JD (2018) Pierre Marie Arthur Morelet (1809–1892) and his Contributions to Malacology. Nederlandse Malacologische Vereniging, Leiden.

[B22] Brown DS (1980) Freshwater Snails of Africa and Their Medical Importance (1^st^ edn.). Taylor and Francis Ltd., London, 487 pp.

[B23] Brown DS (1994) Freshwater Snails of Africa and Their Medical Importance. Revised second edition. Taylor and Francis Ltd., London, 687 pp 10.1201/9781482295184

[B24] Brown DS Kristensen TK (1993) A Field Guide to African Freshwater Snails. 1. West African species. Danish Bilharziasis Laboratory, Charlottenlund, 58 pp.

[B25] Camara IA Bony YK Diomandé D Edia OE Konan FK Kouassi CN Gouréne G Pointier JP (2012) Freshwater snail distribution related to environmental factors in Banco National Park, an urban reserve in the Ivory Coast (West Africa). African Zoology 47: 160–168. 10.3377/004.047.0106

[B26] Cowie RH (2015) The recent apple snails of Africa and Asia (Mollusca: Gastropoda: Ampullariidae: *Afropomus*, *Forbesopomus*, *Lanistes*, *Pila*, *Saulea*): A nomenclatural and type catalogue. The apple snails of the Americas: Addenda and corrigenda, 92 pp. 10.11646/zootaxa.3940.1.1 25947491

[B27] Daget J (2003) Les Mollusques terrestres et fluviatiles du mont Nimba. Mémoires du Muséum national d’histoire naturelle 190: 183–209.

[B28] Danish Bliharziasis Laboratory (1981) Guide de terrain des gastéropodes d’eau douce Africains. 1 Afrique Occidentale. Danish Bilharziasis Laboratory, Charlottenlund.

[B29] Danladi SI Istifanus WA Babayo A (2019) The freshwater snail fauna of the Dadinkowa man-made reservoir, Gombe State, Nigeria. International Journal of Fauna and Biological Studies 6: 31–35.

[B30] Dautzenberg P (1912) Les Annales de l’Institut Océanographique Mission Gruvel sur la còte occidentale d’Afrique (1909–1910): les mollusques marins. In: Joubin L (Ed.) Fondation Albert 1er, Prince de Monaco, 111 pp 10.5962/bhl.title.12889

[B31] Diakité NR Winkler MS Coulibaly JT Guindo-Coulibaly N Utzinger J N’Goran EK (2017) Dynamics of freshwater snails and Schistosoma infection prevalence in schoolchildren during the construction and operation of a multipurpose dam in central Côte d’Ivoire. Infectious Diseases of Poverty 6: 1–93. 10.1186/s40249-017-0305-3 28468667 PMC5415719

[B32] Diomandé D Bony KY Edia EO Konan KF Gourène G (2009) Diversité des Macroinvertébrés Benthiques de la Rivière Agnéby (Côte d’Ivoire; Afrique de l’Ouest). European Journal of Scientific Research 35: 368–377.

[B33] Diomandé D Kpai N Kouadio KN Da Costa SK Gourene G (2013) Spatial distribution and structure of benthic macroinvertebrates in an artificial reservoir: Taabo Lake (Côte d’ Ivoire). International Journal of Biological and Chemical Sciences 7: 1503–1514. 10.4314/ijbcs.v7i4.7

[B34] Dohrn H (1878) Ueber afrikanische Binnenconchylien. Jahrbücher der Deutschen Malakozoologischen Gesellschaft 5: 150–156.

[B35] Durand JR Lévêque C (1981) Flore et Faune Aquatiques de l’Afrique Sahelo-Soudanienne. (Tome 2). ORSTOM, Paris, 410 pp.

[B36] Edokpayi CA Ikharo EA (2011) The Malaco- Faunal characteristics of the “Sandwiched” Epe Lagoon, Lagos. Researcher 3: 15–21.

[B37] Finlayson CM Gordon C Ntiamoa-Baidu Y Tumbulto J Storrs M (2000) The hydrobiology of Keta and Songor lagoons: Implications for coastal wetland management in Ghana. Supervising Scientist Report 152. Darwin.

[B38] Gadzama IMK (2012) Distribution and taxonomy of molluscs (Mollusca) in some parts of northern Nigeria. PhD Thesis, Ahmadu Bello University, Nigeria.

[B39] Gadzama IMK Ezealor AU Aken’Ova T Balarabe ML (2015) Aspects of the Geomorphology and Limnology of some mollusc- inhabited freshwater bodies in northern Nigeria. Journal of Environmental Science, Toxicology and Food Technology 9: 20–29. 10.9790/2402-091112029

[B40] Germain L (1911) Contributions à la faune malacogique de l’Afrique équatoriale. (XXVII). Bulletin du Muséum National d’Histoire Naturelle Paris 17: 319–324.

[B41] Germain L (1917) Contributions à la faune malacologique de l’Afrique équatoriale. XLVII. Mollusques recueillis au Dahomey par M. Henry Hubert. Bulletin du Muséum National d’Histoire Naturelle 23: 511–520.

[B42] Gnohossou PM (2006) La faune benthique d’une lagune ouest africaine (le lac Nokoue au Benin), diversité, abondance, variations temporelles et spatiales, place dans la chaine trophique. PhD thesis, Institut National Polytechnique de Toulouse, France.

[B43] Gofas S Luque ÁA Templado J Salas C (2017) A national checklist of marine Mollusca in Spanish waters. Scientia Marina 81: 241–254. 10.3989/scimar.04543.21A

[B44] Graf DL Cummings KS (2011) Freshwater mussel (Mollusca: Bivalvia: Unionoida) richness and endemism in the ecoregions of Africa and Madagascar based on comprehensive museum sampling. Hydrobiologia 678: 17–36. 10.1007/s10750-011-0810-5

[B45] Gray JE (1847) A list of the genera of recent Mollusca, their synonyma and types. Proceedings of the Zoological Society of London 15: 129–219.

[B46] Greer GJ Mimpfoundi R Malek EA Joky A Ngonseu E Ratard RC (1990) Human schistosomiasis in Cameroon. The American Journal of Tropical Medicine and Hygiene 42: 573–580. 10.4269/ajtmh.1990.42.573 2372088

[B47] Guiral D Albaret J-J Baran E Bertrand F Debenay JP Diouf PS Guillou JJ Le Loeuff P Montoroi J-P Sow M (1999) Rivières du Sud: sociétés et mangroves ouest-africaines (Vol. 1). Editions de l’IRD (ex-Orstom).

[B48] Hayes KA Burks RL Castro-Vazquez A Darby PC Heras H Martín PR Qiu J-W Thiengo SC Vega IA Wada T Yusa Y Burela S Cadierno MP Cueto JA Dellagnola FA Dreon MS Frassa MV Giraud-Billoud M Godoy MS Ituarte S Koch E Matsukura K Pasquevich MY Rodriguez C Saveanu L Seuffert ME Strong EE Sun J Tamburi NE Tiecher MJ Turner RL Valentine-Darby PL Cowie RH (2015) Insights from an Integrated View of the Biology of Apple Snails (Caenogastropoda: Ampullariidae). Malacologia 58: 245–302. 10.4002/040.058.0209

[B49] Hubendick B (1977) Fresh-water gastropods of Sierra Leone. Acta Regiae Societatis Scientiarum et Litterarum Gothoburgensis, Zoologica 11: 1–30.

[B50] Ibikounlé M Satoguina J Fachinan R Tokplonou L Batcho W Kindé-Gazard D Mouahid G Moné H Massougbodji A Courtin D (2013) Épidémiologie de la bilharziose urinaire et des geohelminthiases chez les jeunes scolaires des zones lacustres de la commune de So-Ava, sud-Bénin. Journal of Applied Biosciences 70: 1–5632. 10.4314/jab.v70i1.98805

[B51] Ibikounlé M Massougbodji A Sakiti NG Pointier JP Moné H (2008) Anatomical characters for easy identification between *Biomphalaria pfeifferi*, *Helisoma duryi* and *Indoplanorbis exustus* during field surveys. Journal of Cell and Animal Biology 2: 112–117.

[B52] Ibikounlé M Mouahid G Sakiti NG Massougbodji A Moné H (2009) Freshwater snail diversity in Benin (West Africa) with a focus on human schistosomiasis. Acta Tropica 111: 29–34. 10.1016/j.actatropica.2009.02.001 19426659

[B53] Ibikounlé M Gbédjissi LG Ogouyèmi-Hounto A Batcho W Kindé-Gazard D Massougbodji A (2014a) Schistosomose et géohelminthoses dans le nord-est du Bénin: cas des écoliers des communes de Nikki et de Pèrèrè. Bulletin de la Société de Pathologie Exotique 107: 171–176. 10.1007/s13149-014-0344-y 24595888

[B54] Ibikounlé M Ogouyèmi-Hounto A Sissinto Savi de Tové Y Dansou A Courtin D Kindé-Gazard D Mouahid G Moné H Massougbodji A (2014b) Épidémiologie de la Schistosomose urinaire chez les enfants scolarisés de la commune de Péhunco dans le Nord Bénin: prospection malacologique. Bulletin de la Societe de Pathologie Exotique 107: 177–184. 10.1007/s13149-014-0345-x 24615433

[B55] Imoobe TOT (2008) Variation in benthic macroinvertebrate assemblages in Ologe Lagoon, Nigeria. African Journal of Aquatic Science 33: 45–50. 10.2989/AJAS.2007.33.1.5.389

[B56] Jelnes JE (1986) Experimental taxonomy of *Bulinus* (Gastropoda: Planorbidae): the West and North African species reconsidered, based upon an electrophoretic study of several enzymes per individual. Zoological Journal of the Linnean Society 87: 1–26. 10.1111/j.1096-3642.1986.tb01327.x

[B57] Jørgensen A Kristensen TK Madsen H (2008) A molecular phylogeny of apple snails (Gastropoda, Caenogastropoda, Ampullariidae) with an emphasis on African species. Zoologica Scripta 37: 245–252. 10.1111/j.1463-6409.2007.00322.x

[B58] Jørgensen A Kristensen TK Stensgaard A-S (2010a) *Lanistes libycus* The IUCN Red List of Threatened Species 2010: e.T175137A7110785. 10.2305/IUCN.UK.2010-3.RLTS.T175137A7110785.en

[B59] Jørgensen A Kristensen TK Stensgaard A-S Van Damme D (2010b) *Pila wernei* The IUCN Red List of Threatened Species 2010: e.T175127A7104032. 10.2305/IUCN.UK.2010-3.RLTS.T175127A7104032.en

[B60] Jørgensen A Madsen H Nalugwa A Nyakaana S Rollinson D Stothard JR Kristensen TK (2011) A molecular phylogenetic analysis of *Bulinus* (Gastropoda: Planorbidae) with conserved nuclear genes. Zoologica Scripta 40: 126–136. 10.1111/j.1463-6409.2010.00458.x

[B61] Kittur A Suh B Chi EH (2008) Can you ever trust a wiki?: impacting perceived trustworthiness in wikipedia. In: Proceedings of the 2008 ACM conference on Computer supported cooperative work. ACM, 477–480. 10.1145/1460563.1460639

[B62] Köhler F Glaubrecht M (2006) The types of Ampullariidae Gray, 1824 (Mollusca, Gastropoda) in the malacological collection of the Natural History Museum, Berlin: An annotated catalogue with lectotype designations. Zoosystematics and Evolution 82: 198–215. 10.1002/mmnz.4850820107

[B63] Koné K Bony Kotchi Y Konan Koffi F Edia Oi E Gnagne T (2013) Freshwater snail dynamics focused on potential risk of using urine as fertilizer in Katiola, an endemic area of Schistosomiasis (Ivory Coast; West Africa). Journal of Entomology and Zoology Studies 1: 110–115.

[B64] Kouadio KN Diomandé D Ouattara A Kone YJM Gourene G (2008) Taxonomic Diversity and Structure of Benthic Macroinvertebrates in Aby Lagoon (Ivory Coast, West Africa). Pakistan Journal of Biological Sciences 11: 2224–2230. 10.3923/pjbs.2008.2224.2230 19137831

[B65] Kouadio KN Diomandé D Koné YJM Bony KY Ouattara A Gourène G (2011) Distribution of benthic macroinvertebrate communities in relation to environmental factors in the Ebrié lagoon (Ivory Coast, West Africa). Vie Et Milieu-Life and Environment 61: 59–69.

[B66] Koudenoukpo C (2018) Evaluation de la qualité écologique de la rivière So au Sud Benin: diversité et distribution des assemblages de zooplancton et des macroinvertébrés aquatiques. PhD Thesis, Université d’Abomey-Calavi, Bénin.

[B67] Kristensen TK Ogunnowof O (1987) *Indoplanorbis exustus* (Deshayes, 1834), a freshwater snail new for Africa, found in Nigeria (Pulmonata: Planorbidae). Journal of Molluscan Studies 53: 245–246. 10.1093/mollus/53.2.245

[B68] Kristensen TK Christensen AG (1991) Morphometry versus electrophoresis in *Bulinus* taxonomy–a reply. Journal of Molluscan Studies 57: 299–300. 10.1093/mollus/57.2.299

[B69] Kristensen TK Stensgaard A-S (2010a) *Cleopatra bulimoides*. The IUCN Red List of Threatened Species 2010: e.T175131A7106773.

[B70] Kristensen TK Stensgaard A-S (2010b) *Gabbiella africana* The IUCN Red List of Threatened Species 2010: e.T165403A6017400. 10.2305/IUCN.UK.2010-3.RLTS.T165403A6017400.en

[B71] Kristensen TK Stensgaard A-S (2010c) *Hydrobia accrensis* The IUCN Red List of Threatened Species 2010: e.T165400A6016291. 10.2305/IUCN.UK.2010-3.RLTS.T165400A6016291.en

[B72] Kristensen TK Stensgaard A-S (2010d) *Neritina adansoniana*. The IUCN Red List of Threatened Species 2010: e.T165788A6126163.

[B73] Kristensen TK Stensgaard A-S (2010e) *Neritina oweniana*. The IUCN Red List of Threatened Species 2010: e.T40087A10303057.

[B74] Kristensen TK Stensgaard A-S (2010f) *Potadoma freethi*. The IUCN Red List of Threatened Species 2010: e.T175120A7099504.

[B75] Kristensen TK Stensgaard A-S (2010g) *Segmentorbis angustus*. The IUCN Red List of Threatened Species 2010: e.T165771A6114438.

[B76] Le Loeuff P (1999) La macrofaune d’invertébrés benthiques des écosystèmes à salinité variable le long des côtes atlantiques de l’Afrique tropicale; variations de la biodiversité en relation avec les conditions climatiques actuelles (précipitations) et l’histoire climatique. Zoosystema 21: 557–571.

[B77] Le Loeuff P Zabi GSF (1993) Revue des connaissances sur la faune benthique des milieux margino-littoraux d’Afrique de l’Ouest. Troisième partie: discussion et conclusions. Revue d’Hydrobiologie Tropicale 26: 127–137.

[B78] Lydeard C Cowie RH Ponder WF Bogan AE Bouchet P Clark SA Cummings KS Frest TJ Gargominy O Herbert DG Hershler R Perez KE Roth B Seddon M Strong EE Thompson FG (2004) The Global Decline of Nonmarine Mollusks. BioScience 54: 321–330. 10.1641/0006-3568(2004)054[0321:TGDONM]2.0.CO;2

[B79] Mafiana CF Beyioku YO (1998) *Schistosoma haematobium* infection in Abeokuta. African Journal of Medicine and Medical Sciences 27: 5–7. 10456120

[B80] Mandahl-Barth G (1965) The species of the genus *Bulinus*, intermediate hosts of Schistosoma. Bulletin of the World Health Organisation 33: 33–44. PMC2475805 5294263

[B81] Mandahl-Barth G (1967) Revision of the African genera *Potadoma* Gray and *Potadomoides* Leloup, and description of a new species of *Cleopatra* (GastropodaProsobranchia: Melaniidae). Revue de Zoologie et de Botanique Africaine 76: 110–131.

[B82] Maslin J Bouvet Y (1986) Population Dynamics of *Corbula trigona* (Mollusca) in Lake Ahémé, a West African Lagoon in Benin. Oikos 46: 292–302. 10.2307/3565826

[B83] Meier-Brook C (1983) Taxonomic studies on *Gyraulus* (Gastropoda: Planorbidae). Malacologia 24: 1–113.

[B84] Mimpfoundi R Greer GJ (1990) Allozyme variation among populations of *Biomphalaria pfeifferi* (krauss, 1848) (Gastropoda: Planorbidae) in Cameroon. Journal of Molluscan Studies 56: 461–467. 10.1093/mollus/56.4.461

[B85] MolluscaBase (2019a) [unassigned] Caenogastropoda http://www.molluscabase.org/aphia.php?p=taxdetails&id=382204 [2019-10-08]

[B86] MolluscaBase (2019b) MolluscaBase. http://www.molluscabase.org

[B87] MolluscaBase (2019c) MolluscaBase. *Hydrobia lineata* Jekelius, 1944 †. http://www.molluscabase.org/aphia.php?p=taxdetails&id=822957 [Date 2019-10-10]

[B88] MolluscaBase (2019d) MolluscaBase. *Vitta kuramoensis* (Yoloye & Adegoke, 1977). http://www.molluscabase.org/aphia.php?p=taxdetails&id=1362576 [Date 2019-10-01]

[B89] Nicklès M (1950) 2 Manuels Ouest-Africains Mollusques testacés marins de la Côte occidentale d’Afrique (Vol. 2). Chevalier P (Ed.) Paris, 270 pp.

[B90] Ntonifor HN Ajayi JA (2007) Studies on the ecology and distribution of some medically important freshwater snail species in Bauchi State, Nigeria. International Journal of Biological and Chemical Sciences 1: 121–127. 10.4314/ijbcs.v1i2.39681

[B91] Odountan OH (2017) Ecologie comparée des Macroinvertébrés et Bioindication de la Qualité de l’eau des Lacs Nokoué et Ahémé au Bénin (Afrique de l’Ouest). PhD Thesis, Université d’Abomey-Calavi, Bénin.

[B92] Odountan OH Janssens de Bisthoven L Abou Y Eggermont H (2019a) Biomonitoring of lakes using macroinvertebrates: recommended indices and metrics for use in West Africa and developing countries. Hydrobiologia 826: 1–23. 10.1007/s10750-018-3745-2

[B93] Odountan OH de Bisthoven LJ Koudenoukpo CZ Abou Y (2019b) Spatio-temporal variation of environmental variables and aquatic macroinvertebrate assemblages in Lake Nokoué, a RAMSAR site of Benin. African Journal of Aquatic Science 44: 219–231. 10.2989/16085914.2019.1629272

[B94] Okafor FC Ngang I (2008) Freshwater snails of Niger-Cem, Nkalagu Eastern Nigeria: observations on some demographic aspects of the Schistosome-Transmitting Bulinids. Animal Research International 1: 120–124. 10.4314/ari.v1i2.40754

[B95] Olomukoro JO Azubuike CN (2009) Heavy metals and macroinvertebrate communities in Bottom sediment of Ekpan Creek, Warri, Nigeria. Jordan Journal of Biological Sciences 2: 1–8.

[B96] Oloyede OO Otarigho B Morenikeji O (2017) Diversity, distribution and abundance of freshwater snails in Eleyele dam, Ibadan, south-west Nigeria. Zoology and Ecology 27: 35–43. 10.1080/21658005.2016.1245934

[B97] Onzo-Aboki AA Ibikounlé M Alabi E Dine Mahama S Djenontin A Pennetier C Courtin D (2018) Water quality and dynamic of Schistosomiasis intermediate host snails in the Lake areas, District of So-Ava, Southern Benin. International Journal of Multidisciplinary and Current Research 6: 1390–1398. 10.14741/ijmcr/v.6.6.16

[B98] Ouedraogo I Oueda A Sirima D Ouedraogo I Guenda W Kabre GB (2015) Assessment of benthic mollusc diversity and distribution in urban reservoirs (Ouagadougou, Burkina Faso). International Journal of Biological and Chemical Sciences 9: 2066–2077. 10.4314/ijbcs.v9i4.29

[B99] Ouedraogo I Oueda A Kabore I Moog O Mano K Guenda W Weesie PDM Melcher A Kabre GB (2018) Freshwater molluscs distribution in relation to human activities in the Nakanbé Catchment (Burkina Faso). Journal of Biodiversity and Environmental Sciences 12: 133–145.

[B100] Owojori OJ Asaolu SO Ofoezie IE (2006) Ecology of freshwater snails in Opa reservoir and research farm ponds at Obafemi Awolowo University Ile-Ife, Nigeria. Journal of Applied Sciences 6: 3004–3015. 10.3923/jas.2006.3004.3015

[B101] Özdikmen H Darilmaz MC (2007) *Africanogyrus* nom. n., a replacement name for the preoccupied snail genus *Afrogyrus* Brown & Mandahl-Barth, 1973 (Gastropoda: Planorbidae). African Invertebrates 48: 259–260.

[B102] Pilsbry HA Bequaert JT (1927) The aquatic mollusks of the Belgian Congo. With a geographical and ecological account of Congo malacology. Bulletin of the American Museum of Natural History 53: 69–602.

[B103] Régnier C Fontaine B Bouchet P (2009) Not knowing, not recording, not listing: numerous unnoticed mollusk extinctions. Conservation Biology 23: 1214–1221. 10.1111/j.1523-1739.2009.01245.x 19459894

[B104] Reid DG Dyal P Williams ST (2010) Global diversification of mangrove fauna: a molecular phylogeny of Littoraria (Gastropoda: Littorinidae). Molecular Phylogenetics and Evolution 55: 185–201. 10.1016/j.ympev.2009.09.036 19808097

[B105] Riedel F Von Rintelen T Erhardt S Kossler A (2009) A fossil Potadoma (Gastropoda: Pachychilidae) from Pleistocene central Kalahari fluvio-lacustrine sediments. Hydrobiologia 636: 493–498. 10.1007/s10750-009-9958-7

[B106] Rosewater J (1981) The family Littorinidae in tropical West Africa. Atlantide Report 13: 7–48.

[B107] Salawu OT Odaibo AB (2012) Preliminary study on ecology of *Bulinus jousseaumei* in *Schistosoma haematobium* endemic rural community of Nigeria. African Journal of Ecology 51: 441–446. 10.1111/aje.12054

[B108] Salawu OT Odaibo AB (2014) The bionomics and diversity of freshwater snails species in Yewa North, Ogun State, Southwestern Nigeria. Helminthologia 51: 337–344. 10.2478/s11687-014-0250-7

[B109] Salzburger W Van Bocxlaer B Cohen AS (2014) Ecology and Evolution of the African Great Lakes and Their Faunas. Annual Review of Ecology, Evolution, and Systematics 45: 519–545. 10.1146/annurev-ecolsys-120213-091804

[B110] Sankaré Y Etien N (1991) Analyse des effets de l’ouverture du chenal de Grand Bassam (estuaire du fleuve Comoé, Lagune Ebrié) sur la macrofaune benthique lagunaire. Journal Ivoirien d’Océanologie et de Limnologie 1: 81–90.

[B111] Schultheiß R Van Bocxlaer B Wilke T Albrecht C (2009) Old fossils-young species: Evolutionary history of an endemic gastropod assemblage in Lake Malawi. Proceedings of the Royal Society B: Biological Sciences 276: 2837–2846. 10.1098/rspb.2009.0467 PMC2839958 19439440

[B112] Schultheiß R Van Bocxlaer B Riedel F Von Rintelen T Albrecht C (2014) Disjunct distributions of freshwater snails testify to a central role of the Congo system in shaping biogeographical patterns in Africa. BMC Evolutionary Biology 14: 1–42. 10.1186/1471-2148-14-42 24597925 PMC4015641

[B113] Seddon M Appleton C Van Damme D Graf D (2011) Chapter 4. Freshwater molluscs of Africa: diversity, distribution, and conservation. In: Darwall WKS Allen D Holland R Harrison I Brooks E (Eds) The Diversity of Life in African Freshwaters: Under Water, Under Threat.An Analysis of the Status and Distribution of Freshwater Species Throughout Mainland Africa. IUCN, Gland, Cambridge, 94–125.

[B114] Sellin B Simonkovich E Roux J (1980) Etude de la répartition des mollusques hôtes intermédiaires des schistosomes en Afrique de l’Ouest: premiers résultats. Médecine Tropicale 40: 31–39. 7366365

[B115] Smith KG Diop MD Niane M Darwall WRT (2009) The status and distribution of freshwater biodiversity in Western Africa. Cambridge Publishers, Gland, Switzerland and Cambridge, IUCN, 94 pp. [cover. pp.]

[B116] Strong EE Gargominy O Ponder WF Bouchet P (2008) Global diversity of gastropods (Gastropoda; Mollusca) in freshwater. Hydrobiologia 595: 149–166. 10.1007/s10750-007-9012-6

[B117] Tampo L (2015) Hydrochimie et hydrobiologie du bassin du Zio (Togo): développement d’un indice multimétrique basé sur les macroinvertébrés. PhD Thesis, Université de Lomé, Togo

[B118] Taylor DW (2003) Introduction to Physidae (Gastropoda: Hygrophila); biogeography, classification, morphology. Revista de Biología Tropical 51: 1–287. 15260168

[B119] Van Bocxlaer B Damme D Van Feibel CS (2008) Gradual versus punctuated equilibrium evolution in the Turkana Basin molluscs: Evolutionary events or biological invasions? Evolution 62: 511–520. 10.1111/j.1558-5646.2007.00296.x 17999724

[B120] Van Bocxlaer B Clewing C Etimosundja JM Kankonda A Ndeo OW Albrecht C (2015) Recurrent camouflaged invasions and dispersal of an Asian freshwater gastropod in tropical Africa. BMC Evolutionary Biology 15: 1–33. 10.1186/s12862-015-0296-2 25886047 PMC4373078

[B121] Van Damme D (1984) The Freshwater Mollusca of Northern Africa. Distribution, Biogeography and Palaeoecology. Junk W (Ed.). Dordrecht, Boston, Lancaster.

[B122] Van Damme D Pickford M (2003) The late Cenozoic Thiaridae (Mollusca, Gastropoda, Cerithioidea) of the Albertine Rift Valley (Uganda-Congo) and their bearing on the origin and evolution of the Tanganyikan thalassoid malacofauna. Hydrobiologia 498: 1–83. 10.1023/A:1026298512117

[B123] Van Damme D Ghamizi M Seddon MB Budha PB Dutta J Cordeiro J (2017) *Haitia acuta* The IUCN Red List of Threatened Species 2017: e.T155538A91354457. 10.2305/IUCN.UK.2017-3.RLTS.T155538A91354457.en

[B124] Villanueva C-M (2004) Biodiversité et relations trophiques dans quelques milieux estuariens et lagunaires de l’Afrique de l’Ouest: Adaptations aux pressions environnementales. PhD Thesis, Institut National Polytechnique de Toulouse, France.

[B125] Wanninger A Wollesen T (2019) The evolution of molluscs. Biological Reviews 94: 102–115. 10.1111/brv.12439 PMC6378612 29931833

[B126] Wright CA (1961) Taxonomic problems in the molluscan genus *Bulinus*. Transactions of the Royal Society of Tropical Medicine and Hygiene 55: 225–231. 10.1016/0035-9203(61)90056-6 13786769

[B127] Zabi GSF Le Loeuff P (1992) Revue des connaissances sur la faune benthique des milieux margino-littoraux d’Afrique de l’Ouest. Première partie: biologie et écologie des espèces. Revue d’Hydrobiologie Tropicale 25: 209–251.

[B128] Zabi GSF Le Loeuff P (1993) Revue des connaissances sur la faune benthique des milieux margino-littoraux d’Afrique de l’Ouest. Deuxième partie: peuplements et biotopes. Revue d’Hydrobiologie Tropicale 26: 19–51.

[B129] Zinsou HL (2017) Diversité et écologie des macroinvertébrés benthiques du fleuve Ouémé. PhD Thesis, Université d’Abomey-Calavi, Bénin.

